# Coupling Langevin Dynamics With Continuum Mechanics: Exposing the Role of Sarcomere Stretch Activation Mechanisms to Cardiac Function

**DOI:** 10.3389/fphys.2018.00333

**Published:** 2018-04-06

**Authors:** Takumi Washio, Seiryo Sugiura, Ryo Kanada, Jun-Ichi Okada, Toshiaki Hisada

**Affiliations:** ^1^UT-Heart Inc., Kashiwa, Japan; ^2^Graduate School of Frontier Sciences, University of Tokyo, Kashiwa, Japan; ^3^Predictive Health Team, Integrated Research Group, Compass to Healthy Life Research Complex Program, RIKEN, Kobe, Japan

**Keywords:** multiscale method, Langevin equation, continuum mechanics, actomyosin, heartbeat, stretch activation

## Abstract

High-performance computing approaches that combine molecular-scale and macroscale continuum mechanics have long been anticipated in various fields. Such approaches may enrich our understanding of the links between microscale molecular mechanisms and macroscopic properties in the continuum. However, there have been few successful examples to date owing to various difficulties associated with overcoming the large spatial (from 1 nm to 10 cm) and temporal (from 1 ns to 1 ms) gaps between the two scales. In this paper, we propose an efficient parallel scheme to couple a microscopic model using Langevin dynamics for a protein motor with a finite element continuum model of a beating heart. The proposed scheme allows us to use a macroscale time step that is an order of magnitude longer than the microscale time step of the Langevin model, without loss of stability or accuracy. This reduces the overhead required by the imbalanced loads of the microscale computations and the communication required when switching between scales. An example of the Langevin dynamics model that demonstrates the usefulness of the coupling approach is the molecular mechanism of the actomyosin system, in which the stretch-activation phenomenon can be successfully reproduced. This microscopic Langevin model is coupled with a macroscopic finite element ventricle model. In the numerical simulations, the Langevin dynamics model reveals that a single sarcomere can undergo spontaneous oscillation (15 Hz) accompanied by quick lengthening due to cooperative movements of the myosin molecules pulling on the common Z-line. Also, the coupled simulations using the ventricle model show that the stretch-activation mechanism contributes to the synchronization of the quick lengthening of the sarcomeres at the end of the systolic phase. By comparing the simulation results given by the molecular model with and without the stretch-activation mechanism, we see that this synchronization contributes to maintaining the systolic blood pressure by providing sufficient blood volume without slowing the diastolic process.

## Introduction

With the advances in computational science made possible by improvements in hardware technology, it is now possible to create multi-scale simulation models of the heart in which the macroscopic behaviors of the beating heart can be reproduced and analyzed based on molecular mechanisms of the excitation-contraction coupling process (Kerckhoffs et al., 2007; Gurev et al., 2011; Sugiura et al., 2012). These models are based on many studies of cell models of cardiac electrophysiology (Luo and Rudy, 1994; ten Tusscher et al., 2004; Grandi et al., 2010). We also note that tissue modeling has provided deep insights into the nature of coupling and other interactions among cells in the heart wall (Clayton et al., 2011). Central to these *in silico* heart studies is an accurate model of crossbridge kinetics, which not only forms the basis of cardiac mechanics, but also has clinical relevance in the light of the many reports showing the involvement of sarcomeric proteins in the pathogenesis of cardiomyopathies (Cahill et al., 2013).

Ideally, a molecular dynamics simulation of actomyosin should be coupled with a macroscopic finite element model of the heart because with such a model the impact of mutations in the myosin molecules on cardiac function can be directly assessed. However, it is not possible to perform such simulations even with the best available high-performance computers, and current multi-scale heart simulators usually adopt state-transition models of crossbridge cycling. In these models, the rate constants for transitions between states are governed by the energy of each state (Huxley and Simmons, 1971), but the minimum in the energy landscape corresponding to each state ignores its width in the infinitely-sharp minimum approximation, in which the angle of each lever arm is fixed in the most stable configuration. Obviously, this is a simplification of the behavior of real myosin molecules experiencing thermal fluctuations, and we have recently reported that a model with an energy landscape possessing wide minima can reproduce experimental findings with higher accuracy (Marcucci et al., 2016). However, in that paper, we only examined simple Langevin dynamics with a single variable representing the free energy potential during the power stroke, and solved it using a Monte Carle (MC) simulation. In that case, the Kramers-Smoluchovski approximation (Gardiner, 2004) was used to obtain the rate constants of the transitions between the multiple states, which were given by discretizing the one-dimensional range of the angles of the lever arms. If we try to formulate a more realistic free energy potential as a function of multiple variables, the number of MC states increases explosively, and it is no longer possible to find the rate constants between the MC states theoretically. Therefore, it is desirable to establish a numerical scheme that directly couples the Langevin dynamics of the molecules with the macroscopic continuum dynamics.

Here, we report a novel numerical method to couple the microscale simulation of crossbridge kinetics described by the Langevin equation with the macroscopic mechanics simulations using the finite element method, even though the time scales differ considerably. In this method, the time step of the macroscopic model is set at a multiple of that from the microscopic model to reduce computational overhead. The validity of the method was confirmed with a comparison of the simulation results with the recently reported experimental findings on the spontaneous oscillation of cardiac sarcomeres (Ishiwata et al., 2011), which can be reproduced only by correctly handling the coupling of the motion of the sarcomeres with the actomyosin dynamics. By applying this method, we also show that a trapped crossbridge mechanism greatly facilitates ventricular function through the stretch-activation of the cardiac muscle (Stelzer et al., 2006). A notable feature of the stretch-activation is a long-lasting increase in the contractile tension after a small, rapid stretch is applied during activation. In the usual stretch-activation experiments, the stretch is 1% of the muscle length, which closely corresponds to the microscale size of lever arm swing (10 nm). It is likely that the rapid stretching induces an unusual persistent conformational change of the bound myosin molecules. In this work, we introduce a free energy potential for the power-stroke model in which some of the bound myosin molecules become trapped in a deformed conformation when a rapid stretch is applied. These trapped myosin molecules cannot recover under normal thermal fluctuation unless their rods become relaxed or extremely stretched by subsequent sarcomeric movements. Through the beating-ventricle simulations, we show how this mechanism contributes to improved blood circulation.

## Materials and methods

**Figure 1:**
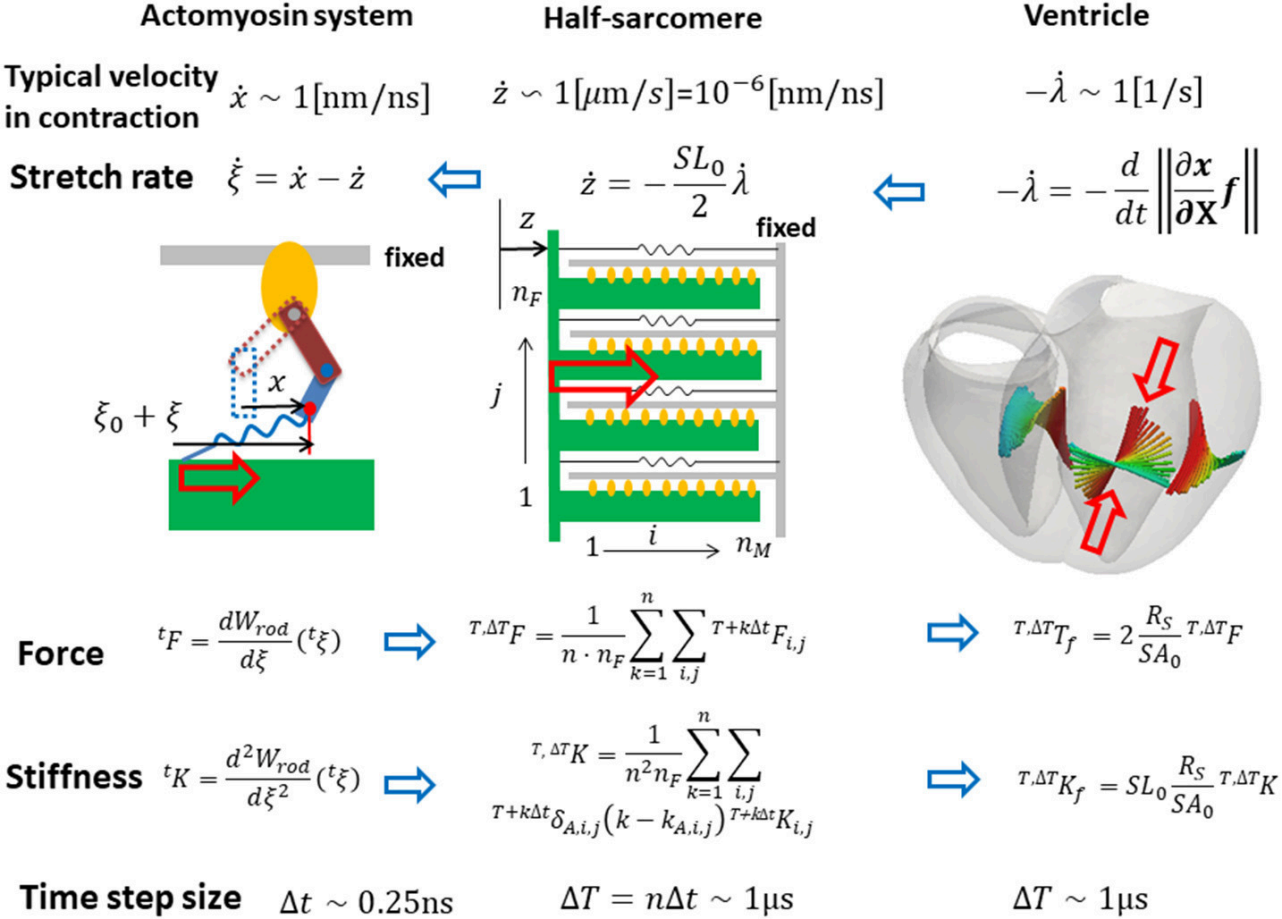
Coupling strategy for three scales. In the actomyosin system, *x* and ξ are variables representing the deformation of the bound myosin molecule. In particular, ξ is the strain of the myosin rods, and *W*_*rod*_(ξ) is its strain energy. These variables were updated by the time step Δ*t* ~ 0.25 ns, while the variables in the half-sarcomere and the ventricle models were updated by the time step Δ*T* = *nΔt* ~ 1 μ*s*. The shortening of the half-sarcomere model is represented by the variable *z*, which affected the Langevin dynamics of the bound myosin molecules through the constraint condition: Δξ = Δ*x* − Δ*z*, while the sarcomeric contractile force ^*T*, Δ*T*^*F* on the time interval [*T, T* + Δ*T*] was given by the sum of tensile forces ^*T*+*k*Δ*t*^*F*_*i,j*_ of the bound myosin rods averaged over the time interval (*k* = 1, ⋯ , *n*). The half-sarcomere model of actomyosin complexes was imbedded into each tetrahedral element of the finite element ventricle model in the reference configuration along the fiber direction ***f***. The deformation at the time *T* of the ventricle is represented by the current position ***x*** = ^*T*^***x***(***X***) of the material point ***X***, thus λ = ||∂^*T*^***x***/∂***X*** · ***f***|| is the stretch along the fiber orientation direction. This stretching was transferred to the shortening of the imbedded half-sarcomere model with the factor −*SL*_0_/2, while the contractile tension *T*, Δ*T T*_*f*_ was given along the fiber direction by scaling the sarcomeric contractile force *T*, Δ*TF* by taking the cross-sectional area per thin filament (*SA*_0_), and the volume ratio of the sarcomere within the ventricle wall (*R*_*S*_) into account.

Our strategy of coupling the different scales is summarized in Figure 1. The stretch rates were transferred from the macro- to micro-scale while the contractile forces were transferred back from the micro- to macro-scale. Finite element continuum mechanics were applied to the ventricle model. The half-sarcomere model of actomyosin complexes was imbedded into each tetrahedral element of the finite element ventricle model along the fiber direction. The molecular variables that represent the deformation of bound myosin molecules were computed by the Langevin dynamics. The shortening rate $-\dot{\lambda }$ along the fiber direction in the ventricle model was transferred to the sarcomeric shortening velocity ż by scaling with the unloaded half-sarcomere length *SL*_0_/2. The sarcomeric shortening velocity ż was applied in the actomyosin model to slide the myosin thick filament. The contractile force of the half-sarcomere model was given by the sum of the tensile forces of the bound myosin rods. The contractile force in the half-sarcomere model was transferred to the macroscopic contractile tension along the fiber direction. In our coupling approach, the computational time step size Δ*T* of the sarcomeric dynamics and the ventricle continuum dynamics is given by an integer multiple of the time step size Δ*t* of the actomyosin Langevin dynamics (Δ*T* = *n*Δ*t*) to reduce the computational and communication overheads. As will be discussed in section Multiple Time Step (MTS) Method, such a multiple time-step strategy can be applied without suffering numerical instabilities by also transferring the stiffness given by the bound myosin rods. Readers who are not interested in the numerical schemes may skip sections Multiple Time Step (MTS) Method and Coupling With the Finite Element Ventricle Model.

### Langevin dynamics of a single sarcomere

**Figure 2:**
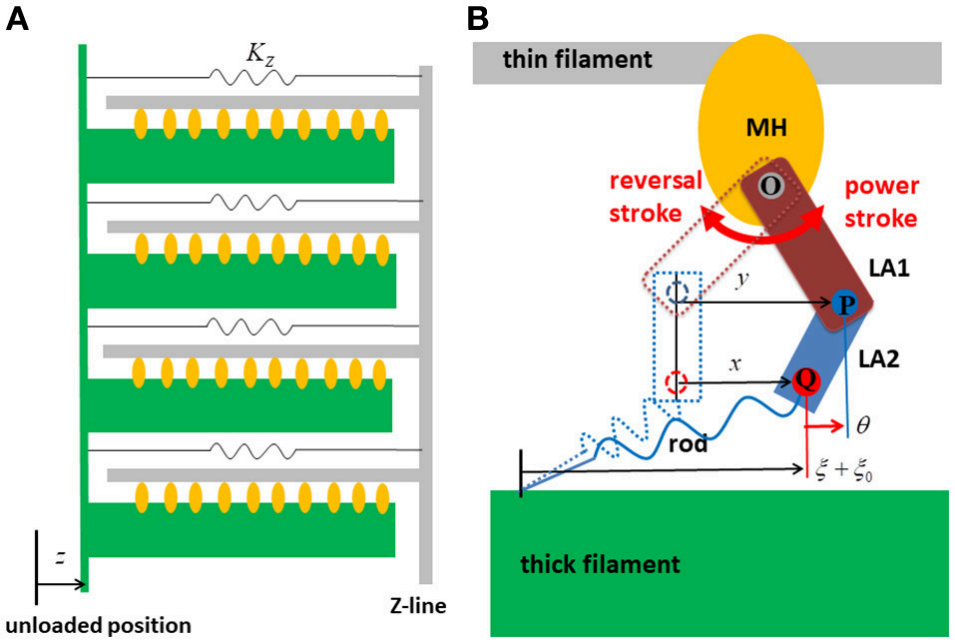
The half sarcomere model **(A)** and the attached myosin molecular model composed of the myosin head (MH) and the lever arm (LA) and the rod **(B)**. In the half-sarcomere model, the Z-line was fixed and the shortening distance of the left edge of the thick filaments is denoted by *z*. The configuration of the attached myosin molecules is represented by two variables *x* and *y*. The LA is decomposed into LA1 and LA2 to represent its deflection around the point “P.” The degree of the deflection is given by θ = *y* − *x*. The power stroke is given by the counter clockwise rotation of LA1 around the point “O.” The rod is a non-linear spring connecting the thick filament and the point “Q” of LA2. The strain of the rod is denoted by ξ.

**Figure 3:**
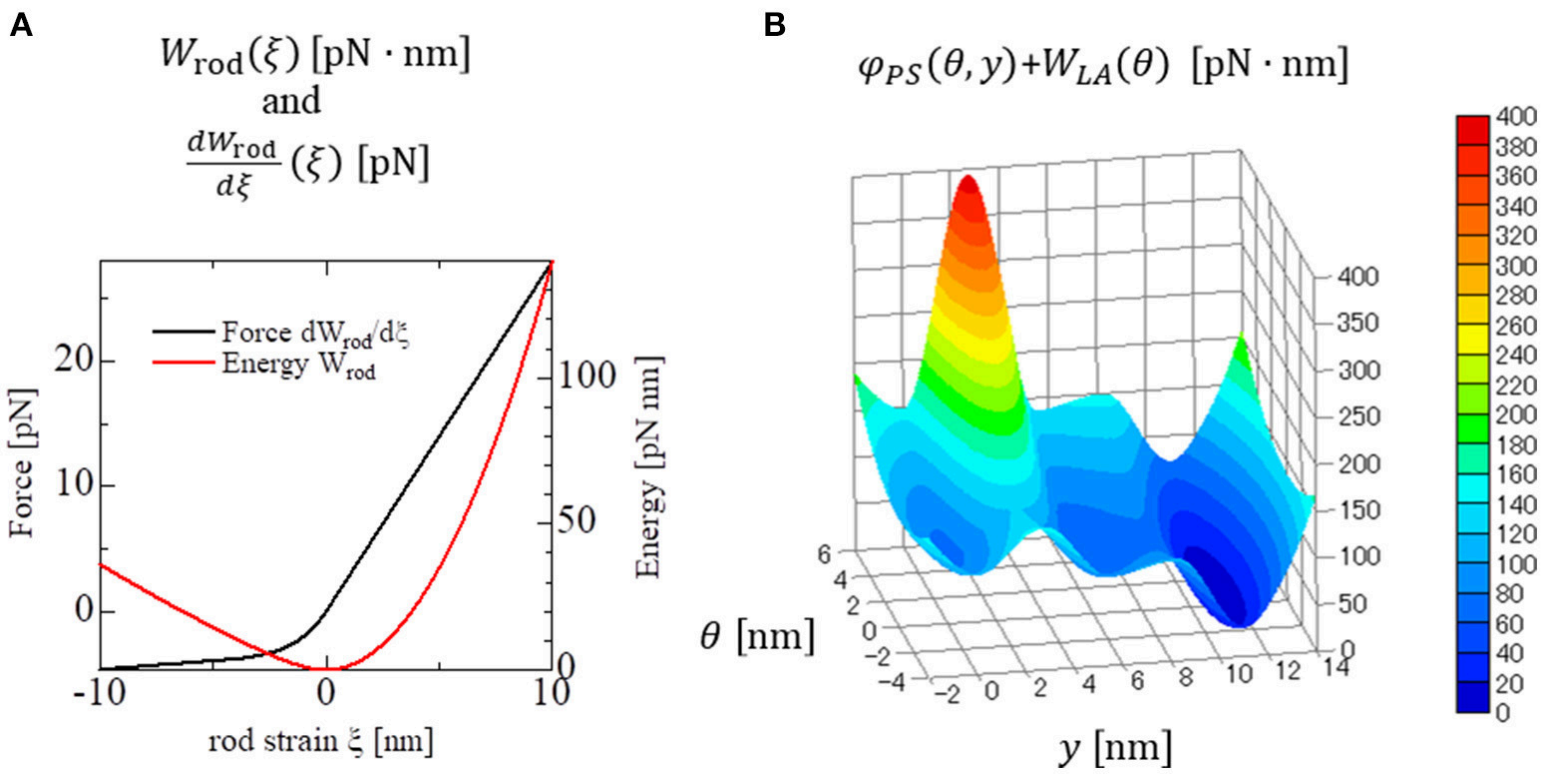
Strain energy *W*_*rod*_(ξ) of the myosin rod and the force given by its derivative **(A)**. The details are described in Supplementary Material S1.2. The free energy landscape of the myosin molecule φ in the attached state with respect to the molecular variables *y* and θ **(B)**. This free energy consists of φ_*PS*_(θ, *y*) and *W*_*LA*_(θ). φ_*PS*_(θ, *y*) is the energy source of the power stroke (rotation of LA1). *W*_*LA*_(θ) is the deformation energy of LA for its deflection. The pre-power stroke configuration corresponds to the local minimum at *y* = 0. The other two local minima at *y* = *s*_1_ = 5.5 nm and at *y* = *s*_1_ + *s*_2_ = 11 nm correspond to the states after the first and second power strokes, respectively. Between the pre-and the post first power stroke states, the high energy barrier is assumed for the positive deflection (θ > 0) of LA.

The parameters adopted for the molecular dynamics are summarized in Table 1. Here, the dynamic equations for a half-sarcomere model composed of *n*_*F*_ pairs of thick and thin filaments (Figure 2A) are introduced. In this half-sarcomere model, we assumed that the right ends of the thin filaments were connected to the Z-line, which was fixed in microscopic space. The shortening displacement of the left end of the thick filament from the unloaded position was denoted by *z*. On each thick filament, there were *n*_*M*_ myosin molecules, which underwent repeated attachment and detachment with the thin filament. The value of *n*_*M*_ = 38 was adopted from our previous work (Washio et al., 2016). During the attached phase, the lever arm (LA) of the myosin molecule rotated around the joint point “O” of the myosin head (MH) under a given free energy potential φ with additional random forces (Figure 2B). These rotations were either the power stroke or the reversal stroke, depending on the rotational direction. To represent the deflection of the LA, it was decomposed into two rigid components, LA1 and LA2, jointed at the point “P” (Figure 2B). As with the real structure of a myosin molecule, LA1 may contain a series of subdomains from the lower 50 kDa to the converter in the motor domain because some conformational changes of these parts were supposed to be accompanied by lever arm rotation. The displacement of the point “P” of the filament direction given by the rotation of LA1 from its pre-power stroke position around the joint point “O” was represented by *y*. Here, the conformation of the myosin molecule just after attachment was assumed to be the same as the pre-power stroke conformation. Similarly, the displacement about the joint point “Q” with the myosin rod was represented by *x*. Thus, θ = *y* − *x* was the deflection of the LA from the pre-power stroke conformation. The strain energy of the myosin rod was given by a function *W*_*rod*_(ξ), where ξ was the strain (length change) in the filament direction from its unloaded natural length ξ_0_. The rod strain energy was non-linear with the generated force, as with our previous work (Washio et al., 2016) for a rod with ξ < 0. For positive strain (ξ > 0), a constant stiffness with a spring constant 2.8 pN/nm was used (Figure 3A). Under these assumptions, the dynamics of the sarcomere was described by the following Langevin equations, where the suffixes *i* and *j* represent the indexes of the MHs and the thick filaments, respectively. Also, *t* is the time, and ^*t*^δ_*A,i,j*_ is set to one if the MH was attached at time *t* to the thin filament, and zero otherwise.

(1) $$\{\begin{array}{l} {\gamma_{X}}^{t}{\dot{x}}_{i,j}+\frac{\partial \varphi }{\partial x}{(}_{\;}^{t}x_{i,j}{,}_{\;}^{t}y_{i,j})+\frac{dW_{rod}}{d\xi }{(}_{\;}^{t}\xi_{i,j}){-}^{t}R_{X,i,j}=0\\ \;{\gamma_{Y}}^{t}{\dot{y}}_{i,j}+\frac{\partial \varphi }{\partial y}{(}_{\;}^{t}x_{i,j}{,}_{\;}^{t}y_{i,j}){-}^{t}R_{Y,i,j}=0{\text{ , }}_{\;}^{t}\delta_{A,i,j}=1(1\leq i\leq n_{M},1\leq j\leq n_{F})\\ {\;}_{\;}^{t}\xi_{i,j}{-}^{t_{A,i,j}}\xi_{i,j}-{(}_{\;}^{t}x_{i,j}{-}^{t_{A,i,j}}x_{i,j}){+}^{t}z{-}^{t_{A,i,j}}z=0 \end{array}$$

(2) γDtξ˙i,j+dWroddξ( tξi,j)−tRD,i,j=0,  δtA,i,j=0(1≤i≤nM,1≤j≤nF)

(3) γZtz˙+KZtz−1nF∑j=1nF∑i=1nMtδA,i,jdWroddξ(ξti,j)=0

**Table 1:** Parameters for the actomyosin dynamics.

**Parameter**	**Value**	**Unit**	**References**
**ATP HYDROLYSIS ENERGY**			
***E*_*ATP*_**	22*k*_*B*_*T*		
***k*_*B*_*T***	4.278	pN · nm	*T* = 310˙C
**POWER STROKE FREE ENERGY φ_*PS*_**			
***s***_1_	5.5	nm	Equation 8
***s***_2_	5.5	nm	Equation 8
***E*_*Pre*_**	0.8*E*_*ATP*_		Equation 8
***E***_***PS***1_	0.85*E*_*Pre*_		Equation 8
***E***_***PS***2_	0.0		Equation 8
***k*_*Y*_**	20	pN/nm	Equation 8
**FOR TRAP MODEL**			
***E***_***b***01_	1.6*E*_*Pre*_		Equation 9
***E***_***b***02_	1.2*E*_*PS*1_		Equation 10
***C*_*trap*_**	200	pN/nm	Equation 9
θ_***trap***_	−0.25	nm	Equation 9
Δθ_***trap***_	5	nm	Equation 9
**FOR NO TRAP MODEL**			
***E***_***b***01_	1.67*E*_*Pre*_		Equation 9
***C*_*trap*_**	0	pN/nm	Equation 9
***E***_***b***02_	1.2*E*_*PS*1_		Equation 10
**ATTACHMENT RATE CONSTANT**			
***A*_*Pre*_**	3,000	1/s	Figure 4
**DETACHMENT RATE CONSTANT TO P_XB_**			
***D***_***PXB**,**Pre***_	3,000	1/s	Equation 11
**DETACHMENT RATE CONSTANT TO N_XB_**			
***D***_***NXB***0_	125	1/s	Equation 12
***a*_*min*_**	0.1	1/nm^2^	Equation 13
***c*_*min*_**	100	1/s	Equation 13
***d*_*min*_**	−16	nm	Equation 13
***a*_*max*_**	0.1	1/nm^2^	Equation 13
***c*_*max*_**	100	1/s	Equation 13
***d***_***max**,**Pre***_	5	nm	Equation 14
***d***_***max**,**PS***1_	9	nm	Equation 14
***d***_***max**,**PS***2_	9	nm	Equation 14
**DAMPING COEFFICIENT**			
γ_***X***_	20	pN ns/nm	Equation 1
γ_***Y***_	50	pN ns/nm	Equation 1
γ_***D***_	70	pN ns/nm	Equation 2

Here, the probabilistic rules for transitions between the attached and detached states will be given below. At the time of attachment, the myosin molecule was assumed to be in the pre-power stroke state.

(4) {xtA,i,ji.j=xpre≡0ytA,i,ji.j=ypre≡0

Here, *t*_*A,i,j*_ is the time at which the attachment occurred. The spring strain *^t^*ξ*_i,j_* was continuously updated at the transitions.

In Equations (1–3), γ_*X*_, γ_*Y*_, and γ_*D*_ were the damping coefficients, and *^t^R_X,i,j_*, *^t^R_Y,i,j_*, and *^t^R_D,i,j_* were the random forces, which fulfilled the condition:

(5) {〈Rα,i,jt〉=0〈Rα,i,jt,t′Rβ,k,l〉=δαβδikδjl2γαkBT     t−t′δ,                                                                     α,β=X,Y,D,     1≤i,k≤nM,1≤j,l≤nF

where Boltzmann's constant is *k*_*B*_ and the temperature is *T*. In this paper, the damping coefficient γ_*D*_ was set to 70 pN · ns/nm, following Howard (2001), while γ_*X*_ and γ_*Y*_ were set to 20 and 50 pN · ns/nm, respectively. Since the rotation of LA1 may involve structural changes in other parts in the MH, the drag coefficient for LA1 was larger than that for LA2.

In Equation (3), γ_*Z*_ was the drag coefficient per length change of a single thin filament of the sarcomere and *K*_*Z*_ was the spring constant for each thin filament of the sarcomere. Equation (3) follows from the fact that the sarcomeric contractile tension is just the sum of the tensile forces of the rods for all of the attached myosin molecules. The third line in Equation (1) indicates the constraint condition in the association state. This condition gives the rod strain ^*t*^ξ_*i,j*_ in relation to the conformational change of the myosin (*^t^x_i,j_*) and the sarcomeric movement (*^t^z*).

### Free energy of a myosin molecule

We assume that the free energy of the myosin molecule φ in the attached state can be decomposed into the power stroke free energy φ_*PS*_ of LA1 and the deflection energy of the LA:

(6) $$\begin{array}{l} \varphi \left(x,y\right)=\varphi_{PS}\left(\theta ,y\right)+W_{\text{LA}}\left(\theta \right),\;\theta =y-x \end{array}$$

For the deflection energy of the LA, a simple quadratic potential was assumed:

(7) $$\begin{array}{l} W_{\text{LA}}\left(\theta \right)=\frac{1}{2}K_{\theta }\theta^{2} \end{array}$$

Since there was no appropriate reference for setting the stiffness, a comparable stiffness (*K*_θ_ = 4 pN/nm) to that of the rod strain was adopted in our model. For the power-stroke free energy φ_*PS*_, the three local minima at *y* = 0, *s*_1_, and *s*_1_ + *s*_2_ for a fixed deflection θ = *y* − *x* are given as shown in Figure 2B, which is described by the following equations:

(8) $$\begin{array}{l} \varphi_{PS}(\theta ,y)=\\ \{\begin{array}{ll} E_{Pre}+\frac{1}{2}(E_{b1}(\theta )-E_{Pre})(1-2\pi \frac{y\;+\;s_{1}/4}{s_{1}})+\frac{1}{2}k_{Y}{\left(y+s_{1}/4\right)}^{2}, & \;y\leq -\frac{s_{1}}{4}\\ E_{Pre}+\frac{1}{2}(E_{b1}(\theta )-E_{Pre})(1-\cos2\pi \frac{y}{s_{1}}), & -\frac{s_{1}}{4}<y\leq \;\frac{s_{1}}{2}\\ E_{PS1}+\frac{1}{2}(E_{b1}(\theta )-E_{PS1})(1-\cos2\pi \frac{y\;-\;s_{1}}{s_{1}}), & \frac{s_{1}}{2}<y\leq s_{1}\;\\ E_{PS1}+\frac{1}{2}(E_{b2}(\theta )-E_{PS1})(1-\text{cos2}\pi \frac{y\;-\;s_{1}}{s_{2}}), & s_{1}<y\leq s_{1}+\;\frac{s_{2}}{2}\\ E_{PS2}+\frac{1}{2}(E_{b2}(\theta )-E_{PS2})(1-\cos2\pi \frac{y\;-\;s_{1}\;-\;s_{2}}{s_{2}}), & s_{1}+\;\frac{s_{2}}{2}<y\leq s_{1}+\frac{5s_{2}}{4}\\ E_{PS2}+\frac{1}{2}(E_{b2}(\theta )-E_{PS2})(1+2\pi \frac{y\;-\;s_{1}\;-\;5s_{2}/4}{s_{2}})+\frac{1}{2}k_{Y}{\left(y-s_{1}-\frac{5s_{2}}{4}\right)}^{2}, & y>\frac{5s_{2}}{4} \end{array}. \end{array}$$

Here, *E*_*Pre*_, *E*_*PS*1_and *E*_*PS*2_ were the three local minimum energy values at *y* = 0, *s*_1_, and *s*_1_ + *s*_2_, respectively. These local minima correspond to the configurations of the MH and LA1 in the pre-power stroke state, and the states after the first two power strokes. The power stroke step sizes, *s*_1_ and *s*_2_, and the energies *E*_*Pre*_ − *E*_*PS*1_ and *E*_*PS*1_ − *E*_*PS*2_ consumed in the two strokes, are given values (Table 1) similar to those used in the Monte Carlo (MC) model in our previous work (Washio et al., 2016), in which the ATP hydrolysis energy was set to *E*_*ATP*_ = 22*K*_*B*_*T* following Saupe et al. (1999) at a body temperature of *T* = 310 *K*.

In Equation (8), *E*_*b*1_(θ) and *E*_*b*2_(θ) are the energy barriers between the minima. The heights of the energy barriers were adjusted so that enhanced beating performance was realized in the coupled simulation for the ventricle model, which is introduced below. In our model, the first barrier was assumed to be a function of the LA deflection θ as:

(9) $$\begin{array}{l} E_{b1}\left(\theta \right)=\{\begin{array}{l} E_{b01},\;\theta \leq \theta_{trap}\\ E_{b01}+C_{trap}\frac{\theta -\theta_{trap}}{\Delta \theta_{trap}},\theta_{trap}<\theta \leq \theta_{trap}+\Delta \theta_{trap}\\ E_{b01}+C_{trap},\;\theta >\theta_{trap}+\Delta \theta_{trap} \end{array} \end{array}$$

This first energy barrier was introduced to reproduce the stretch-activation of the cardiac muscle (Stelzer et al., 2006). In their experiment, a small, rapid stretch of ~1% of the sample length was imposed to activated skinned myocardium. Then, a nearly 10% increase in the contractile tension persisted for a time on the order of seconds compared with that of the steady state before the stretch. This suggests the existence of a trapped conformation for the MH and LA in an attached state that can be generated by the rapid stretch. By experiencing a high barrier, as in Equation (9), a myosin molecule that exhibits a large deflection θ and a large strain ξ after the first power stroke can become trapped in that state if the MH is strongly attached, since these myosin molecules cannot make progress toward a larger forward stroke, which would requires a large increment in either the deflection energy of the LA [*W*_*LA*_(θ)], or the strain energy of the rod [*W*_*rod*_(ξ)]. Such large LA deflections and rod strains can be generated when the thick filament was pulled rapidly to the outside. In this work, the values *C*_*trap*_ = 200 *pN*/nm, θ_*trap*_ = −0.25 nm, and Δθ_*trap*_ = 5 nm were adopted (Figure 3B) so that the appropriate response to the stretch-activation is reproduced, as shown in the numerical simulation. The second energy barrier was assumed to be a constant:

(10) $$\begin{array}{l} E_{b2}\left(\theta \right)\equiv E_{b02}. \end{array}$$

### Control model of attachment and detachment

**Figure 4:**
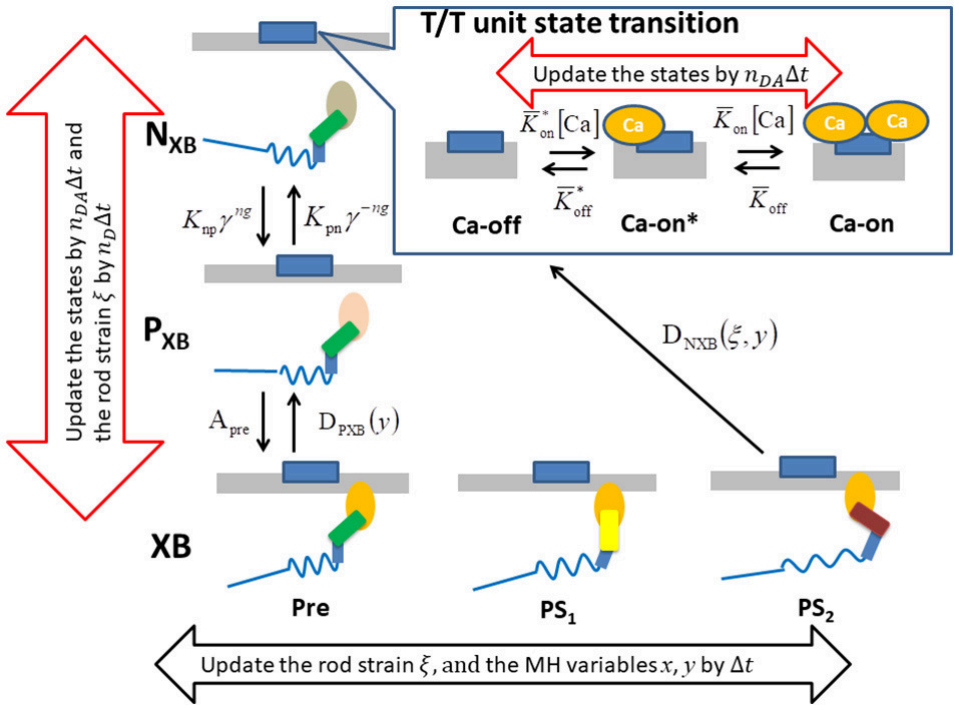
The state transition Monte Carlo model of the T/T unit and the myosin molecule. The MHs in either the N_XB_ or P_XB_ states are assumed to be detached. The rate constant factors *K*_*np*_ and *K*_*pn*_ between N_XB_ and P_XB_ are affected by the state of T/T unit above it. The detachment rate constant *D*_*PXB*_ between P_XB_ and XB are given as a function of *y*, so that the transition to P_XB_ is allowed only for the MHs in the pre-power stroke position. The detachment rate constant *D*_*NXB*_ to N_XB_ is given as a function of *y* and ξ, so that the detachment is allowed for the second post-power stroke state or the MHs connected to the extremely stained myosin rods. The time-step strategy for reducing the computational loads is shown by the arrows. The molecular variables *x*, *y*, and ξ of the bound myosin molecules are updated by the finest basic time step Δ*t* (black arrow), while the rod strain ξ in the detached states is updated by its multiples *n*_*D*_Δ*t*. The state transitions of the MHs and the T/T units are calculated by the MC method with the time step *n*_*DA*_Δ*t* (red arrows).

For the transition between the attached and detached states (Figure 4), an MC model similar to the one in our previous work (Washio et al., 2016) was used. In the half-sarcomere model, the MHs were arranged on the thick filament at regular intervals, and the thin filament was divided into segments called troponin/tropomyosin (T/T) units. The transitions between the states of a T/T unit were affected by the *Ca*^2+^ concentration, [Ca], and by the states of the MHs below the T/T unit. In this model, only the Ca-bound state increased the affinity of the MHs for the thin filament. There are two detached state of MHs - a nonbinding state *N*_*XB*_, and a weakly binding state *P*_*XB*_. The affinity was adjusted by modifying the factor *K*_*np*_ for the rate constant of the transition from *N*_*XB*_ to *P*_*XB*_. The relationship between the *MH*_*i,j*_ location and the T/T unit was determined from the offset position of the MH_*i,j*_ (zt+ξti,j−xti.j) from its unloaded position (Figure 2A). A cooperative mechanism with the nearest-neighbor MHs was added by introducing the factors γ^*ng*^ and γ^−*ng*^ (γ = 40), as in our previous work (Washio et al., 2016), in which the integer *ng* (= 0, 1 *or* 2) was the number of neighboring MHs in the weakly binding state *P*_*XB*_ or the attached state *XB*. The details of the transients of the T/T unit states and between *N*_*XB*_ and *P*_*XB*_ are described in Supplementary Material S1.1.

Attachment was possible only from state *P*_*XB*_ with the rate constant *A*_*Pre*_. Detachment from the attached state *XB* to the weakly bound state *P*_*XB*_ was allowed only from the pre-power stroke state, as follows:

(11) $$\begin{array}{l} D_{PXB}\left(y\right)=\{\begin{array}{ll} D_{PXB,Pre},\; & y\leq s_{1}/4\\ \left(1-\omega \right)D_{PXB,Pre}, & y=(1/4+\omega /2)\;s_{1}:0<\omega \leq 1\\ 0, & y>3s_{1}/4 \end{array} \end{array}$$

Here, the variable ω could take values between 0 and 1, and was introduced to interpolate the rate constant between the pre-power stroke state and the state after the first power stroke.

In this transition, no ATP molecules were consumed, whereas detachment to *N*_*XB*_ required one ATP molecule. This rate constant is given as a function of both the rod strain ξ and the power stroke displacement *y*:

(12) $$\begin{array}{l} D_{NXB}\left(\xi ,y\right)=\\ \{\begin{array}{ll} \max\left(0,D_{strain}\left(\xi ,y\right)\right), & y\leq s_{1}+s_{2}/4\\ \max\left(\omega D_{NXB0},D_{strain}\left(\xi ,y\right)\right), & y=s_{1}+\left(1/4+\omega /2\right)\\ \; & s_{2}:0<\omega \leq 1\\ \max\left(D_{NXB0},D_{strain}\left(\xi ,y\right)\right), & y>s_{1}+3s_{2}/4 \end{array} \end{array}$$

Similar to before, the variable ω could take values between 0 and 1, and interpolated the rate constant between the states after the first and second power strokes, while *D*_*strain*_ indicates the forced detachment due to the extreme strain of the myosin rod:

(13) $$\begin{array}{l} D_{strain}\left(\xi ,y\right)=\\ \{\begin{array}{ll} c_{min}\left(\exp\left(a_{min}{\left(\xi -d_{min}\right)}^{2}\right)-1\right), & \xi \leq d_{min}\\ 0, & d_{min}<\xi \leq d_{max}\left(y\right)\\ c_{max}\left(\exp\left(a_{max}{\left(\xi -d_{max}\left(y\right)\right)}^{2}\right)-1\right), & \xi >d_{max}\left(y\right) \end{array} \end{array}$$

Here, the negative strain threshold *d*_*min*_ was a constant, and the positive strain threshold *d*_*max*_ depended on the stroke displacement *y* as

(14) $$\begin{array}{l} d_{\max}\left(y\right)=\{\begin{array}{ll} d_{\text{max},\text{Pre}}, & y\leq s_{1}/4\\ \left(1-\omega \right)d_{\text{max},\text{Pre}}+\omega d_{\text{max},\text{PS1}}, & y=\left(14+\omega /2\right)\\ \; & s_{1}:0<\omega \leq 1\\ d_{\text{max},\text{PS1}}, & s3_{1}/4<y\leq \;s_{1}+s_{2}/4\\ \left(1-\omega \right)d_{\text{max},\text{PS1}}+\omega d_{\text{max},\text{PS2}}, & y=s_{1}+\left(1/4+\omega /2\right)\\ \; & s_{2}:0<\omega \leq 1\\ d_{\text{max},\text{PS2}}, & y>s_{1}+3s_{2}/4 \end{array} \end{array}$$

In this study, the parameters *d*_*max,Pre*_ = 5 nm, *d*_*max,PS1*_ = 9 nm, and *d*_*max,PS2*_ = 9 nm were used. These values were adjusted so that the appropriate responses to stretch-activation were reproduced. These choices did not conflict with the fact that the binding affinity to the thin filament increased as the power stroke proceeded (Llinas et al., 2015).

### Multiple time step (MTS) method

First, we consider a multiple time step (MTS) approach for a single half-sarcomere model (Figure 2) in which different time step intervals Δ*t* and Δ*T* were adopted, respectively, for updating the molecular variables *x*_*i,j*_, *y*_*i,j*_, ξ_*i,j*_ and the sarcomeric shortening displacement *z*, when solving Equations (1, 2) coupled with Equation (3). Below, this approach will be extended to coupling with a macroscopic finite element continuum model, in which a single sarcomere model was imbedded into each finite element.

The time step Δ*T* was assumed to be an integer multiple of the time step interval for the molecular variables Δ*t*:

(15) $$\begin{array}{l} \Delta T=n\cdot \Delta t \end{array}$$

Such approaches reduce the computational overhead of the shared-memory synchronization, as well as the data communication needed in distributed parallel systems, if a sufficiently large integer *n* can be applied. For our Langevin dynamics model, the microscale time step *t* was set at 0.25 ns. This choice was constrained by the relationships between the magnitudes of the drag coefficients γ_*X*_, γ_*Y*_ with the curvature of the potential φ. For example, in the case of a simple Langevin equation:

(16) γ     tu˙+dφdu(ut)−Rt=0

with a given free energy potential φ, a variable *u*, and the random force that satisfies

(17) {〈Rt〉=0〈RtRt′〉=2γkBTt−t'δ

The stability of the explicit numerical integration scheme required that

(18) $$\begin{array}{l} \Delta t\leq \frac{\gamma }{K_{max}} \end{array}$$

where *K*_*max*_ was the maximum magnitude of the curvature of φ (||*d*^2^φ/*du*^2^||) over the range of *u*. Even if an implicit time integration scheme was applied, Equation (18) must be satisfied for the maximum magnitude value of the negative curvature (*d*^2^φ/*du*^2^ < 0). In our case, as shown in Figure 3B, negative curvatures were unavoidable on the ridge lines of the potential landscape. For example, if Δ*t* = 0.25 ns was used when γ = 50 pN · ns/nm, the allowable maximal curvature from Equation (18) was *K*_*max*_ = γ/Δ*t* = 200 pN/nm. This curvature value implies an energy change of $K_{max}\Delta u^{2}=100\text{ pN}\cdot \text{nm}$ for a displacement Δ*u* = 1 nm. Actually, values for the magnitude of the curvature were observed near the high energy barrier between the pre-power stroke state and the state after the first power stroke in our model (Figure 3B).

Another limitation on practicable time step size comes from considerations of fluctuations Δ*u* during each time interval Δ*t*. If we ignore the potential φ in Equation (16), the standard deviation of Δ*u* given by a series of random forces in Equation (17) during time Δ*t* is

(19) σΔt=〈Δu2〉=2kBTΔt/γ

At body temperature, we have *k*_*B*_*T* = 4.278 *pN* · nm. Thus, for the case of γ = 50 *pN* · *ms*/nm and Δ*t* = 0.25 ns, we have ^Δ*t*^σ ~ 0.2 nm. These displacements are large enough to make a noticeable difference in the landscape of the potential φ.

Compared with the dynamics of the molecules, the sarcomeric movement in cardiac muscle is generally much slower, as shown by the following argument. The shortening velocity of the sarcomere model is related to the stretch rate $\dot{\lambda }$ of the cardiac muscle along the fiber direction by

(20) $$\begin{array}{l} ż=-\frac{1}{2}SL_{0}\dot{\lambda } \end{array}$$

Here, *SL*_0_ = 2.1 μm is the unloaded sarcomere length. If we assume the maximal shortening velocity of the cardiac muscle ${\left(-\dot{\lambda }\;\right)}_{max}=5ML/s$, where ML is the muscle length (Edman et al., 1974), the maximal shortening velocity of a half-sarcomere is ${ż}_{\max}=5.25\mu \text{m}/s=5.25\times 10^{-6}\text{nm}/ns$. However, the previous consideration regarding the fluctuations during the time interval Δ*t* = 0.25 ns gives the average magnitude of the molecular velocity to be ^Δ*t*^σ/*t* ≈ 0.8 nm/*ns*. This comparison between the sarcomeric and molecular velocities suggests the possibility of applying a multi-valued time step approach, in which tens of thousands of fine time steps of size Δ*t* are calculated when integrating the molecular variables *x*_*i,j*_, *y*_*i,j*_, ξ_*i,j*_ for each large one step interval Δ*T* used for integrating the sarcomeric variable *z*.

During the time integration process using the small time step Δ*t* over the time interval [*T* : *T* + Δ*T*], the LA conformation variables *x*_*i,j*_, *y*_*i,j*_ are updated explicitly, and then the rod strains ξ_*i,j*_ are temporarily updated so that the constraint in Equation (1) is fulfilled, using the most recently calculated shortening velocity ż from time *T*. The temporarily updated variables are denoted with bars over them, such as ${\bar{\xi }}_{i,j}$ and $\bar{z}$. When the process switches to an implicit computation of the sarcomeric shortening displacement *z* and its time derivative ż at time *T* + Δ*T* for use in Equation (3), the tensile forces exerted by the attached MHs during the time interval [*T* : *T* + Δ*T*] are computed by using the corrected rod strain ξ_*i,j*_, for which the shortening velocity ż over the time interval [*T* : *T* + Δ*T*] is replaced with ^*T*+Δ*T*^ż. By doing so, the stiffness due to the strained rods of the attached MHs is involved in the implicit time integration of Equation (3). This implicit strategy allows us to apply a time interval Δ*T* which is four orders of magnitude larger than Δ*t*.

The molecular variable time integrations can be performed using the temporal sarcomeric shortening displacement $\bar{z}$ on the time interval [*T* : *T* + Δ*T*] given by

(21) z¯T+ΔT=zT+kΔt    Tz˙,    k=1,⋯,n

The LA conformation variables for the attached MHs at time *t* + Δ*t* are explicitly updated from those at time *t*, so that the following equations are satisfied:

(22) {γXxt+ti.j−xti.jΔt+∂φ∂x(xti.j,yti.j)+dWroddξ(ξ¯ti,j)                                                              −RtX,i,j=0,       δtA,i,j=1γYyt+ti.j−yti.jΔt+∂φ∂y(xti.j,yti.j)−RtY,i,j=0

Then, the temporal rod strains $\left\{{\bar{\xi }}_{i,j}\right\}$ at time *t* + Δ are updated according to

(23) {γXξ¯t+ti.j−ξ¯ti.jt+dWroddξ(ξ¯ti.j)−RtD,i,j=0,            δtA,i,j=0ξ¯t+ti.j−ξ¯tA,i,ji.j−(xt+Δti.j−xtA,i,ji.j)               δtA,i,j=1       +z¯t + Δt−z¯tA,i,j=0,

After performing the above time integrations for *k* = 1, ⋯ , *n* over the interval [*T* : *T* + Δ*T*], the true sarcomeric shortening displacement *z* is implicitly computed by solving the following equations:

(24) {γZT+ΔTz˙+KZT+ΔTz−    T,ΔTF=0zT+ΔT=zT+ΔTz˙T+ΔT

In Equation (24), the mean total tensile force ^*T*,Δ*T*^*F* over the time interval [*T* : *T* + Δ*T*] is found by applying the true rod strains {ξ_*i,j*_} over the time interval [*T* : *T* + Δ*T*] according to

(25) FT,ΔT=F¯T,ΔT+1n · nF∑k=1n∑j=1nF∑i=1nMδT+kΔtA,i,jd2Wroddξ2(ξ¯T+kΔti,j)(ξT+kΔti,j−ξ¯T+kΔti,j)

where the temporary total tensile force is evaluated using

(26) F¯T,ΔT=    1n · nF∑k=1n∑j=1nF∑i=1nMδT+ktA,i,jdWroddξ(ξ¯T+kΔti,j)

from the temporary rod strain values {ξ¯T+kΔti,j}. Note that Equation (25) is a linear approximation of the tensile force for the true rod strains about the temporary rod strains, for which the differences are given by

(27) ξT+kΔti,j−ξ¯T+kΔti,j=−(k−kA,i,j)Δt(z˙T+ΔT−z˙T), k=1,…,n

where *k*_*A,i,j*_ is the most recent microscale step index for *k* for which *MH*_*i,j*_ is attached. This number is initialized to zero before starting the small time steps with *k* = 1. By substituting Equation (27) into Equation (25), the mean total tensile force can be rewritten as

(28) FT,ΔT=F˜T,ΔT−ΔTT,ΔTKFz˙T+ΔT

with total mean stiffness

(29) KT,ΔTF=1n2 · nF∑k=1n∑j=1nF∑i=1nMδT+kΔtA,i,j(k−kA,i,j)                      ×d2Wroddξ2(ξ¯T+kΔti,j)

and extrapolated mean total tensile force using ^*T*^ż

(30) F˜T,ΔT=F¯T,ΔT+ΔTT,ΔTKFz˙T

By substituting Equations (28–30) into Equation (24), the implicit scheme is established as follows:

(31) (γZ+KZΔT+ΔTT,ΔTKF)T+ΔTz˙=−(KZzT−F˜T,ΔT)

To see the necessity of the above implicit coupling scheme, consider the instability of the usual explicit scheme here. If an explicit scheme for the total mean tensile force is used

(32) {γZT+ΔTz˙+KZT+ΔTz−    T,ΔTF¯=0zT+ΔT=zT+ΔTz˙T+ΔT

instead of Equation (24), the time step size Δ*T* is limited by the total mean stiffness by

(33) ΔT<γZ+ΔTKZKT,ΔTF

As an illustration, in the case of $\gamma_{Z}=10^{4}\text{ pN}\cdot \text{ns}/\text{nm}$, as assumed in our previous work (Washio et al., 2017), ^*T*,Δ*T*^*K_F_* = 28 pN/nm, 20 attached MHs, the stiffness of each rod set to 2.8 pN/nm, and *K*_*Z*_ ≈ 0, the constraint in Equation (33) would be Δ*T* < 360 ns. However, the proposed algorithm is stable for any time step size, as far as the linear approximation in Equation (28) is concerned.

In coupling with the macroscopic finite element model, a half-sarcomere model is assigned to each element, for which Equation (20) is applied based on the relationship between the stretching along the fiber orientation **f** and the deformation gradient tensor:

(34) λT=‖∂    Tx∂Xf‖

Here, ^*T*^***x*** = ^*T*^***x***(***X***) is the current position at time *T* of the material point ***X*** in the unloaded condition. Specifically, the following equation, obtained from Equation (34), is substituted into Equation (20).

(35) λ˙T=1λT(∂    Tx˙∂Xf)·(∂    Tx∂Xf)

From Equations (20, 28), the mean total tensile force of each thin filament is given by

(36) FT,ΔT=F˜T,ΔT−ΔTKT,ΔTF2SL0λ˙T+ΔT

Here, ^*T*^ż in Equation (30) is also replaced with −*SL*_0_*^T^*$\dot{\lambda }/2$ to determine F˜T,ΔT. Thus, the total active tension per unit area in the unloaded configuration, the nominal stress, is given by

(37) TT,ΔTf=2RsSA0FT,ΔT=2RsSA0(F˜T,ΔT−ΔTKT,ΔTF2SL0λ˙T+ΔT)

Here, *SA*_0_ is the cross-sectional area of a single thin filament and *R*_*S*_ denotes the volume ratio of the sarcomere. The factor of two in Equation (37) comes from the fact that FT,ΔT is the total tensile force given by the MHs surrounding one of the double spirals along the thin filament.

Although a small time step on the order of 1 ns must be used for the time integration of the molecular variables, a larger time step can be applied to the MC state-transition phase. Thus, it is reasonable to apply a much larger time step size, as long as it is an integer multiple of Δ*t*, to the computation of the MC state-transitions. Furthermore, even for the time integration of the molecular variables, a coarser time step than the one used for the attached MHs can be applied to the detached MHs, since the magnitudes of the curvatures are different for the potentials *W*_*rod*_ and φ (Figure 3).

### Coupling with the finite element ventricle model

**Figure 5:**
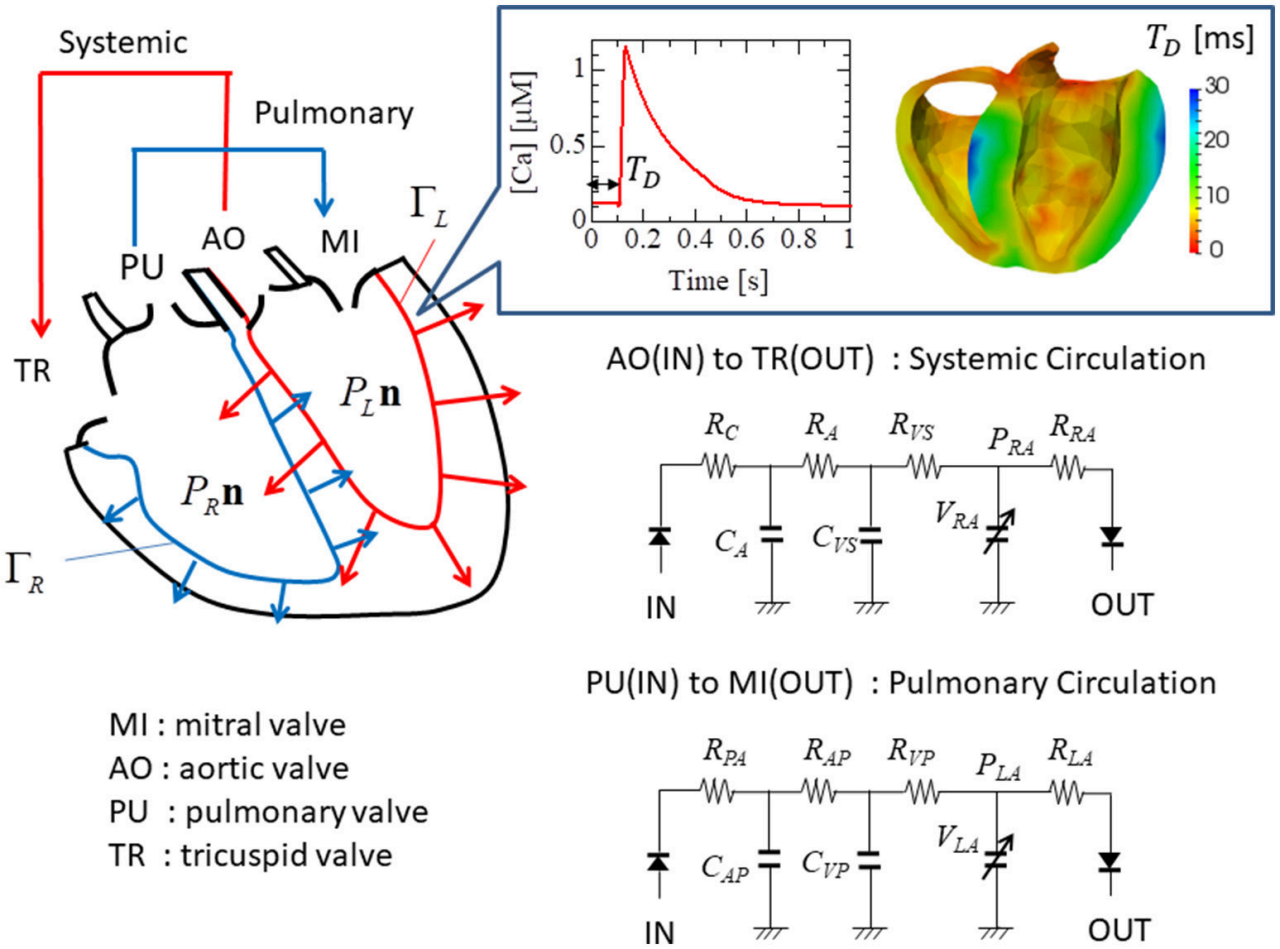
Electrical analog circuits connected to the valve interfaces. The systemic circulation model represents blood flow from the left to the right ventricle through the aortic (AO) and the tricuspid (TR) valves, while the pulmonary circulation model represents blood flow from the right to the left ventricle through the pulmonary (PU) and the mitral (MI) valves. The boxed inset shows the Ca^2+^ transient profile with the delay time *T*_*D*_.

In the beating-ventricle simulation, the Ca^2+^ transient is given for each element of the ventricle model (Figure 5). By referencing the Ca^2+^ transients, together with the stretching λ and the stretching rate $\dot{\lambda }$ along the fiber direction, the molecular variables were integrated using the small time step Δ*t*, while the macroscopic displacements of the continuum were computed using the large time step Δ*T*. As derived in the Supplementary Material S3, the active stress on the continuum at time *T* + Δ*T* is represented by the first Piola–Kirchhoff stress tensor:

(38) Πact= TT,ΔTfT+ΔTλf⊗f·(∂    T+ΔTx∂X)T

In the definition of the tension TT,ΔTf in Equation (37), the stiffness due to the attached MHs is implicitly included by the use of λ˙T+ΔT for z˙T+ΔT in Equation (28). See also the explanation of the stiffness in the Supplementary Material S3. Thus, the proposed scheme is stable for any size of time step.

The governing equation in the macroscale to be solved can be represented by

(39) $$\begin{array}{l} \int_{\Omega }^{\;}\delta \dot{u}\cdot \rho \ddot{u}\;d\Omega +\int_{\Omega }^{\;}\delta \dot{\text{Z}}:{\left(\Pi +2pJF^{-1}\right)}^{T}\;d\Omega \\ \;=\;P_{L}\int_{\Gamma_{L}}^{\;}\delta \dot{u}\cdot n\;d\Gamma_{L}+P_{R}\int_{\Gamma_{L}}^{\;}\delta \dot{u}\cdot n\;d\Gamma_{R} \end{array}$$

(40) $$\begin{array}{l} \int_{\Omega }^{\;}\delta p\left(2\left(J-1\right)-\frac{p}{\kappa }\right)\;d\Omega =0 \end{array}$$

Here, ***u*** = ^*T*^***u***(***X***)=^*T*^***x***(***X***)−***X*** is the displacement of the material at point ***X*** ∈ Ω at time *T*, ρ is the density of the heart muscle, ***F*** = ∂**x**/∂***X*** is the deformation gradient tensor, **Z** = ∂***u***/∂***X*** is the displacement gradient tensor, *J* = det ***F*** is the Jacobian, *p* is the hydrostatic pressure, κ is the bulk modulus, and *P*_*L*_ and *P*_*R*_ are the blood pressures in the left and right ventricles, respectively. Ω is the muscle domain in the reference configuration, while Γ_*L*_ and Γ_*R*_ are the blood–muscle interfaces of the left and right ventricles, respectively, in the current configuration at time *T*, and ***n*** is the normal unit vector directed from the cavity to the muscle at these surfaces (Figure 5). The Dirichlet boundary condition ^***T***^***u***(***X***)=0 is imposed on the boundary nodes around the valve rings. The first Piola–Kirchhoff stress tensor *Π* consists of the active, passive, and viscous stresses:

(41) $$\begin{array}{l} \Pi =\Pi_{act}+\Pi_{pas}+\Pi_{vis} \end{array}$$

where *Π*_*act*_ is given by Equation (38), and the others are, respectively, the passive and viscous stresses, as described in our previous work (Washio et al., 2016). The details of these two stress tensors are given in the Supplementary Material S4.

The ventricle blood pressures *P*_*L*_ and *P*_*R*_ were determined through their interactions with the circulatory system of the body. These were modeled as electrical analog circuits, using the same parameters described in our previous work (Washio et al., 2016). The details of the circuit model that includes the atrial model are given in the Supplementary Material S5. In particular, the flow rates at the inlets and the outlets were associated with the rates of volume change in the cavity according to:

(42) $$\begin{array}{l} \{\begin{array}{l} \int_{\Gamma_{L}}\dot{\text{u}}\cdot \text{n}\;d\Gamma_{L}=F_{MI}-F_{AO}\\ \int_{\Gamma_{R}}\dot{\text{u}}\cdot \text{n}\;d\Gamma_{R}=F_{TR}-F_{PA} \end{array} \end{array}$$

Here, *F*_*MI*_, *F*_*AO*_, *F*_*TR*_, and *F*_*PA*_ were the flow rates, respectively, through the mitral, aortic, tricuspid, and pulmonary valves (Figure 5). These flow rates were determined by Ohm's law while taking the rectification of the valve into account.

(43) $$\begin{array}{l} F=H\left(\bar{F}\right)\bar{F} \end{array}$$

Here, $\bar{F}$ was the flow rate in the case of no rectification, and *H* was the relaxed Heaviside function:

(44) $$\begin{array}{l} H\left(\bar{F}\right)=\{\begin{array}{ll} 0, & \bar{F}<0\\ {\left(\bar{F}/{\bar{F}}_{0}\right)}^{2}\left(3-2\bar{F}/{\bar{F}}_{0}\right), & 0\leq \bar{F}\leq {\bar{F}}_{0}\;\\ 1, & \bar{F}>{\bar{F}}_{0} \end{array} \end{array}$$

In our simulation, the value ${\bar{F}}_{0}=5\text{ mL}/\text{s}$ was used.

The macroscopic variables, including the acceleration $\ddot{u}$, velocity $\dot{u}$, and displacement ***u*** at time *T* + Δ*T* were found using Newton–Raphson iteration until the equilibrium condition was satisfied with the Newmark-beta time integration scheme (Supplementary Material S6). During the iterations, the active stress in Equation (38) was redefined with Equation (37), in which the microscopic computational results F˜T,ΔT and KT,ΔTF were reused. Thus, switching between computations at the two scales only happened once for each macroscopic time step.

## Results

### Computer system

To perform the simulations, a distributed parallel system was used. Each node consisted of two Intel® Xeon® E5-2670 (20 MB Cache, 2.6 GHz) processors, and each processor was composed of 8 cores. In the single sarcomere simulations, only one node was used for shared memory OpenMP parallelization. In the beating-ventricle simulations, the elements of the ventricle wall were equally distributed to the nodes, while the macroscopic computations were performed only at the master node. In the microscopic computations, the time integrations of the molecular variables were parallelized using OpenMP by dividing the filaments equally among the 16 cores.

### Validation of the MTS scheme via single sarcomere oscillation

The accuracy and computational efficiency of the MTS scheme were validated by numerical experiments with a single half-sarcomere model, in which 48 thin filaments were connected to a common Z-line (Figure 2A). In our previous work (Washio et al., 2017), we showed that the spontaneous oscillatory behavior of the sarcomere (Ishiwata et al., 2011) can be explained by the power stroke principle after applying a simple ordinary differential equation model. In this case, the collective reversal power strokes induced quick sarcomeric lengthening. Here, we show that this could also be reproduced by the Langevin dynamics model, regardless of the choice of macroscale time step size in the MTS scheme. In this numerical experiment, the spring constant *K*_*Z*_ was set to 1 pN/nm per thin filament, and the viscosity coefficient γ_*Z*_ was set to 10^4^ pN · ns/nm per thin filament. During the simulations, the Ca^2+^concentration was kept at the constant value of 1 μM.

**Figure 6:**
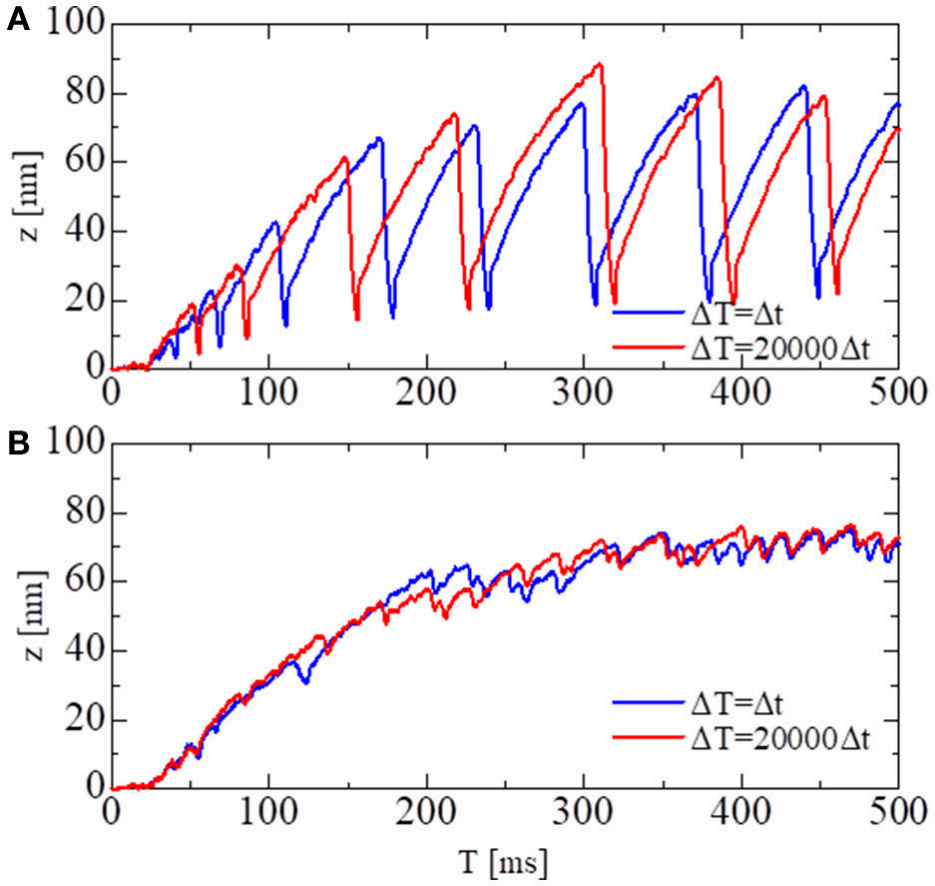
The shortening displacements obtained by the standard scheme (blue: Δ*t* = Δ*T* = 0.25 ns) and the MTS scheme (red: Δ*t* = 0.25 ns, Δ*T* = 5, 000 ns) for the spontaneous oscillations of the single half-sarcomere model with *n*_*M*_ = 38 and *n*_*F*_ = 48. **(A)** The comparison for the no-trap model. **(B)** The comparison for the trap model.

In Figure 6, the shortening displacements obtained by using a conventional single-scale integration scheme (Δ*t* = Δ*T* = 0.25 ns) and the MTS scheme (Δ*t* = 0.25 ns, Δ*T* = 5, 000 ns) are compared for both the no-trap and trap models. In the no-trap model, the dependence of the first energy barrier height *E*_*b*1_(θ) on the LA deflection θ in Equation (9) was eliminated, and the baseline of the energy barrier *E*_*b*01_ was higher when compared with the one in the trap model (Table 1), so that a similar maximal tensile force is obtained in both models. Next, the state-transitions were computed with Δ*t* = 0.25 ns. In these numerical experiments, the simulations started from an initial state in which all of the MHs were in N_XB_, and an identical series of random forces and pseudorandom numbers for the MC state-transitions were applied to all the simulations. In case of the no-trap model (Figure 6A), similar amplitudes and periods were obtained for the shortening displacements, although there were deviations in the timing of the sharp declines. In case of the trap model (Figure 6B), the large dips in the displacements disappeared. Instead, rapid small vibrations appeared. In this case, similar initial rises, periods, and amplitudes of vibrations were obtained for the both time step sizes of Δ*T*.

As depicted in Figure 4, the attached MHs in the *XB* state were classified according to their power stroke displacement *y*, as follows:

(45) $$\begin{array}{l} \{\begin{array}{l} \text{Pre}\;=\;\left\{{\text{MH}}_{i,j}\in \text{XB}:\;y_{i,j}<s_{1}/2\right\}\\ {\text{PS}}_{1}\;=\;\;\{{\text{MH}}_{i,j}\in \text{XB}:\;s_{1}/2\leq y_{i,j}<s_{1}+s_{2}/2\}\\ {\text{PS}}_{2}\;=\;\left\{{\text{MH}}_{i,j}\in \text{XB}:y_{i,j}\geq s_{1}+s_{2}/2\right\} \end{array} \end{array}$$

**Figure 7:**
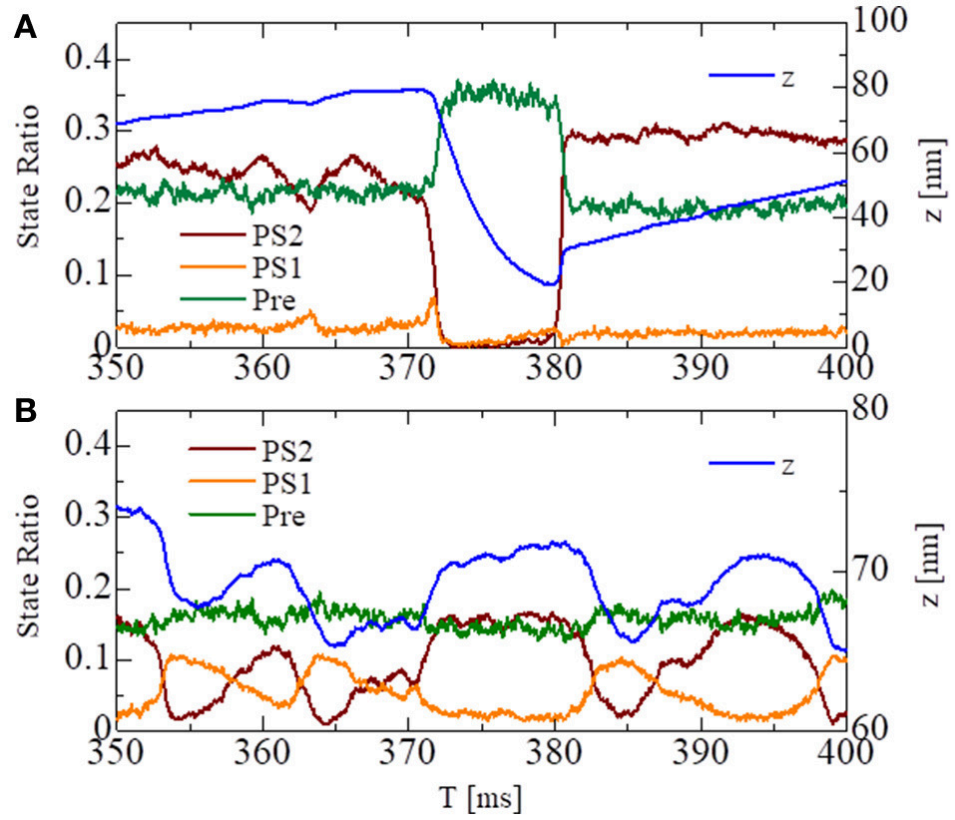
The temporary change of the state ratios classified to the three power stroke stages (*Pre*, *PS*_1_, and *PS*_2_) obtained by the standard scheme (Δ*T* = 0.25 ns) for the spontaneous oscillations of the single half-sarcomere with the no-trap model **(A)** and the trap model **(B)**. In case of the no-trap model **(A)**, a large pulsed flux of the reversal power strokes from *PS*_2_ to Pre through *PS*_1_ generates the sharp decline of *z* around *T* = 370 ms. In case of the trap model **(B)**, the flux of the reversal power strokes is trapped at PS_1_, so that the decline of *z* is stopped within small changes Δ*z* > −10 nm.

These states can be regarded as the pre-power stroke, the state after the first power stroke, and the state after the second power stroke, respectively. As suggested by our previous work (Washio et al., 2017), a large pulsed flux of the reversal power strokes from *PS*_2_ to *Pre* over *PS*_1_ generated the sharp decline in *z* for the no-trap model (Figure 7A). In the trap model, this reversal flux was trapped at *PS*_1_, so that the decline in *z* was stopped at small changes, leading to Δ*z* > −10 nm (Figure 7B), which corresponds to the stroke size of the LA.

**Figure 8:**
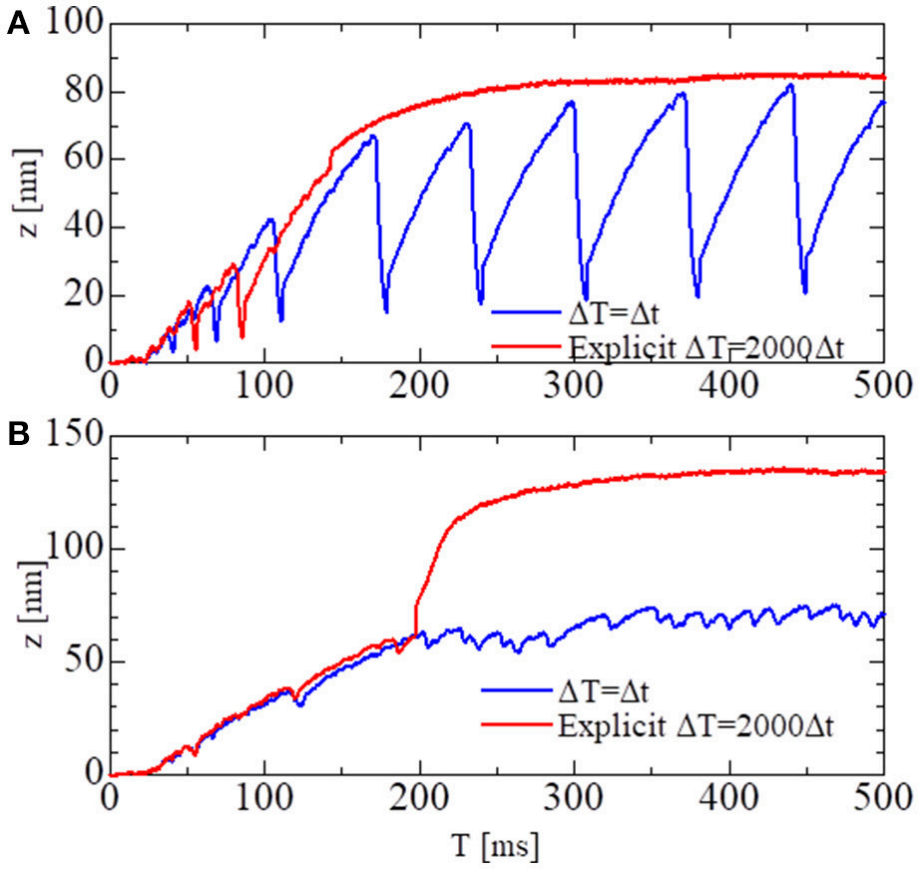
The shortening displacements obtained by the standard scheme (blue: Δ*T* = 0.25 ns) and the explicit scheme (red: Δ*T* = 500 ns) for the spontaneous oscillations of the single half-sarcomere model with *n*_*M*_ = 38 and *n*_*F*_ = 48. **(A)** The comparison for the no-trap model. **(B)** The comparison for the trap model.

To test the stability of the MTS scheme, simulations using the explicit scheme given by Equation (32) were performed with a much smaller time step of Δ*T* = 500 ns (Figure 8). Although the explicit scheme also yielded good results at first, the computational results became totally invalidated when the active stiffness KT,ΔTF exceeded the threshold indicated by Equation (33), as estimated previously. Furthermore, oscillatory behavior could not be reproduced with the explicit scheme. This result suggests the drawback of explicitly using the active tensions, which occurs when solving a system of ordinary differential equations with a finer time step in coupled simulations. As shown in Figure 8A, the calculated force using the explicit scheme did not diverge, although the oscillatory behavior was completely lost. Thus, it is difficult to judge the accuracy of numerical results by examining only one case. As shown here, we must compare the results of different macroscale time step sizes Δ*T* to confirm the accuracy of the coupling scheme.

The above simulations were executed on one node consisting of 16 cores using shared memory in OpenMP parallelization. Thus, in the parallelization, three filaments were assigned to each core. The averaged elapsed times for the 1-ms time integration were 125 and 97 s, with the standard integration scheme (Δ*t* = Δ*T* = 0.25 ns) and the MTS scheme (Δ*t* = 0.25 ns, Δ*T* = 5, 000 ns), respectively. The difference in the elapsed times came from the machine synchronization overhead, and the differences in the computational loads for the various filaments. With the MTS scheme that lumps 20,000 steps, the differences in computational loads between the filaments during each small time step were tremendously diminished. For a single-sarcomere simulation, using a much smaller time step size for Δ*T* was sufficient to attain good parallel efficiency because the overhead associated with updating *z* was negligible. However, a large step size was necessary when the sarcomere model was coupled with the macroscopic ventricle model because the communication overhead between the large number of nodes became greatly increased, along with the computation time for updating the macroscopic variables.

### Validation of basic sarcomere properties

**Figure 9:**
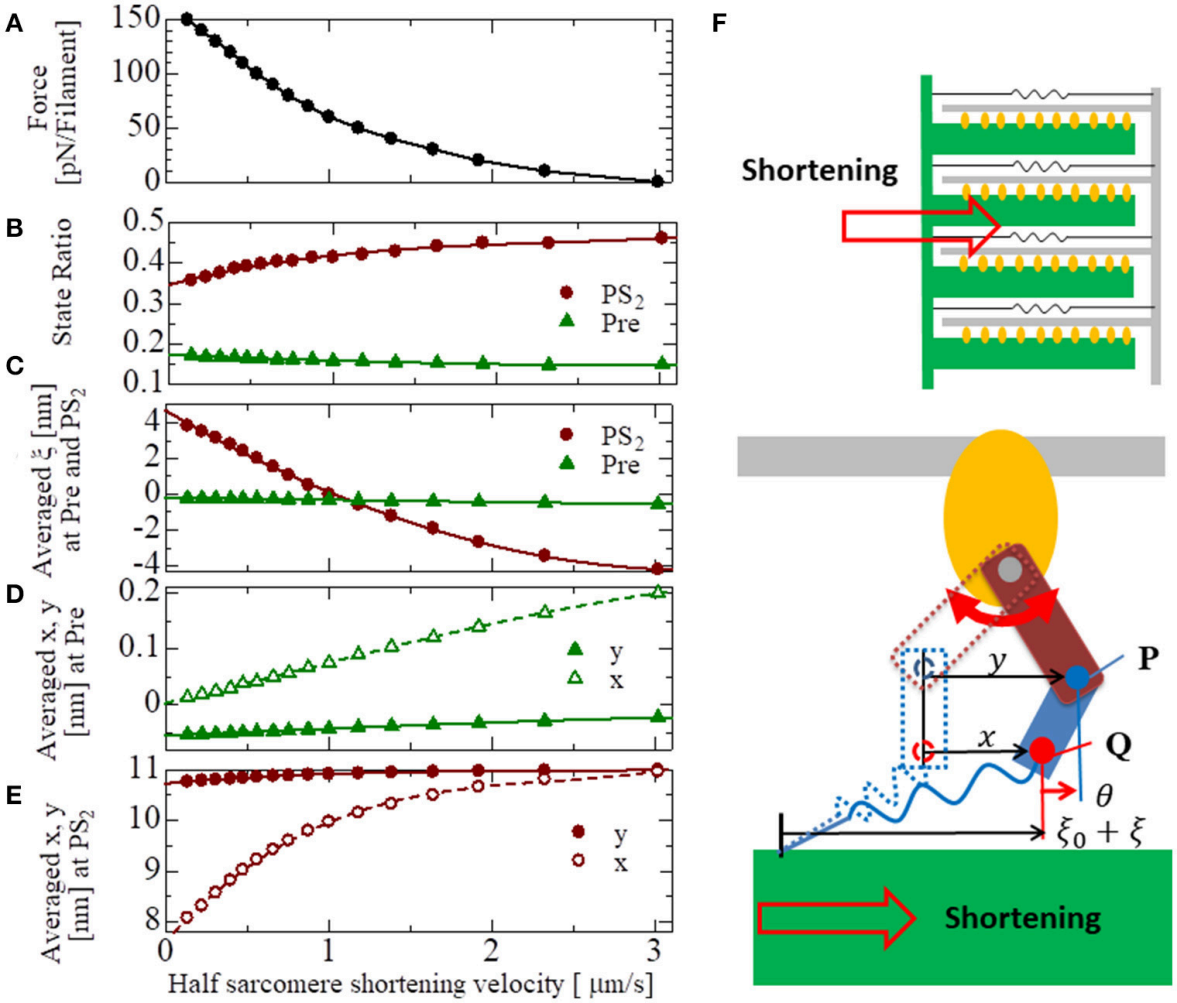
The force-velocity relation for [Ca] = 1 μM with the shortening velocity of the half-sarcomere in the horizontal axis **(A)**. The state ratios of the pre-power stroke (*Pre*) and the post-second power stroke state (*PS*_2_) are plotted with respect to the shortening velocities **(B)**. Similarly, the averaged rod strains ξ in the states *Pre* and *PS*_2_ **(C)**, the averaged molecular variables *x* and *y* of the molecular deformation **(F)** in the states *Pre* and *PS*_2_ are also plotted, respectively, in **(D,E)**.

The basic properties of the actomyosin trap model, which includes the SL and [Ca] dependences of the contractile force, the isometric twitch, the responses for the isotonic contraction, and the quick shortening of the half-sarcomere, along with the details of these numerical experiments, are presented in the Supplementary Material S2. The results of these numerical experiments confirm the validity of our half-sarcomere model. Here, the force-velocity curve obtained at a constant Ca^2+^ concentration ([Ca] = 1 μ*M*) is examined in context with the behavior of the bound myosin molecules during the isotonic contractions at the various shortening velocities (Figure 9). As the shortening velocity increased, the state ratio of *PS*_2_ increased (Figure 9B), because the joint point P was pushed forward (*y* increased) more strongly by the deflection potential *W*_*LA*_(θ) in Equation (7) with the larger negative deflection θ = *y* − *x* (Figure 9D). Note that the negative averaged rod strain ξ at *PS*_2_ for a shortening velocity larger than 1 μm/s (Figure 9C) does not imply a negative contractile force, because *dW*_*rod*_/*dξ*(ξ) ≫ −*dW*_*rod*_/*dξ*(−ξ) for any positive strain ξ>0, except for ξ~0 as shown in Figure 3A.

### Stretch-activation by trapped myosins

**Figure 10:**
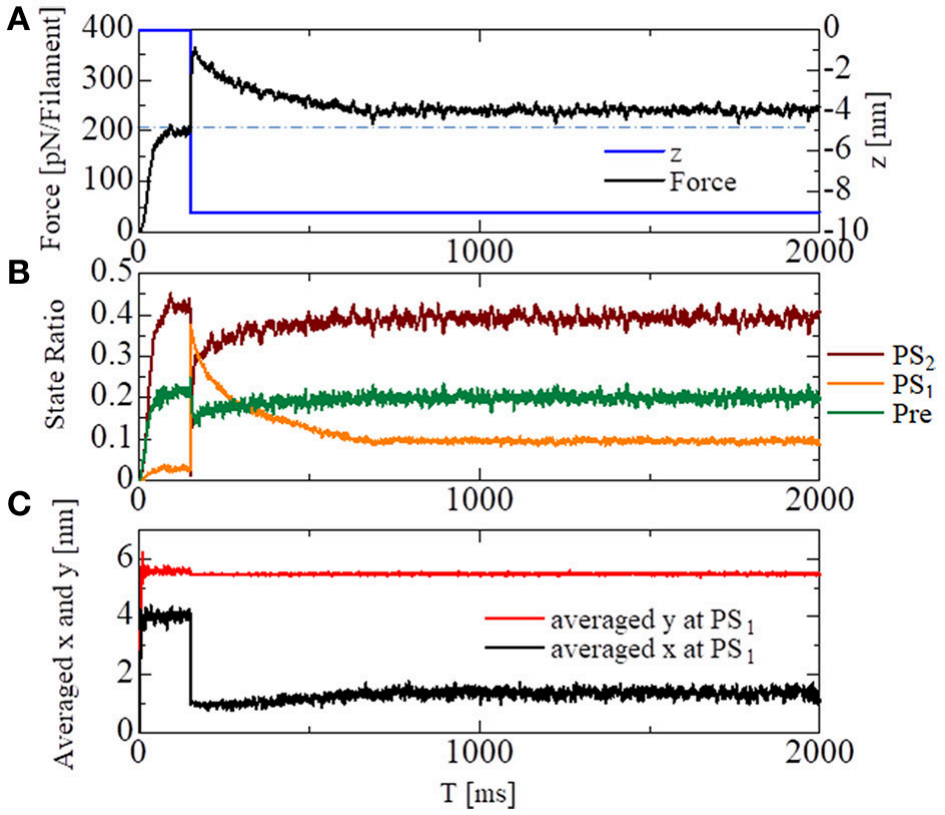
Numerical results of the stretch-activation simulation. A shift of Δ*z* = −9 nm was applied over the time span of 1 ms at *T* = 150 ms. **(A)** The time courses of the contraction force per one thin filament (black) and the applied shortening displacement *z* (blue) are shown. The dot-dash line indicates the baseline of the force before the stretch. **(B)** The state ratios for the three power stroke stages of the attached MHs. **(C)** The averaged molecular variables *x* and *y* for the MHs at *PS*_1_.

To see the effectiveness of the trapping mechanism in the state after the first power stroke *PS*_1_ created by the energy barrier in Equation (9), together with the zero detachment rates for *PS*_1_ in Equations (11, 12), a stretch-activation test was performed for the single half-sarcomere model consisting of 48 filament pairs (Figure 10). Here, a 1% stretch was applied over the 1-ms time interval starting at *T* = 150 ms, at which time the contractile force had sufficiently matured. In the simulation, the time step sizes were set at Δ*t* = 0.25 ns and Δ*T* = 25 ns. The state-transitions were also computed using Δ*t* = 0.25 ns. During the simulations, the Ca^2+^ concentration ([Ca]) was kept at the constant value of 10 μM.

A roughly 15% increase in the contractile force lasted at least 2 s after the quick stretch (Figure 10A). This long-lasting increase in the force compared with the pre-stretch steady state was apparently due to the lasting increase in the population of PS_1_ (Figure 10B: orange line). The persistent increase of the averaged LA deflection, θ = *y* − *x*, for MHs in *PS*_1_ (Figure 10C) indicates that it was generated by the MHs trapped by the higher free energy barrier *E*_*b*1_(θ) defined by Equation (9). Compared with the experimental results given in Stelzer et al. (2006), our numerical result misses “Phase 2,” in which the force drops one time to the steady state level before the stretch. However, the magnitude of the force incrementation after that agrees with the experimental facts.

### Beating-ventricle simulations

Beating-ventricle simulations were performed using a finite element ventricle model consisting of 7,600 tetrahedral elements. In each element, a sarcomere model consisting of 8 filament pairs was imbedded along the appropriate fiber orientation **f**. The distribution of the fiber orientations (Figure 1) was found by an optimization algorithm (Washio et al., 2016) based on the impulses given by the active tension, which was computed using the MC crossbridge model instead of the Langevin model to reduce the heavy computational loads. Portions of the helical fiber structure are depicted in Figure 1. As confirmed in our previous work (Washio et al., 2016), this algorithm constructed a fiber distribution that was quite similar to the one obtained by diffusion tensor magnetic resonance imaging (DTMRI) measurements. The heart rate was set to 60 beats per minute, and the Ca^2+^ transient (Figure 5) generated by the mid-myocardial cell model proposed by ten Tusscher and Panfilov (2006) was applied. The transmural delays of the Ca^2+^ transient determined by the distances from the endocardial surfaces of the left and right ventricles under a transmural conduction velocity of 52 cm/s, as measured by Taggart et al. (2000), was adopted. The deformation of each element was linked to the sarcomeric shortening displacement using Equations (34, 35). In the simulations, the optimized time step algorithm represented in Figure 4 was applied. Essentially, the values Δ*t* = 0.25 ns and *T* = 5, 000 ns were used, so that *n* = 20, 000. However, the state-transitions were computed every 2.5 ns (*n*_*DA*_ = 10), and the time integration for the detached MHs were performed every 1.25 ns (*n*_*D*_ = 5).

**Figure 11:**
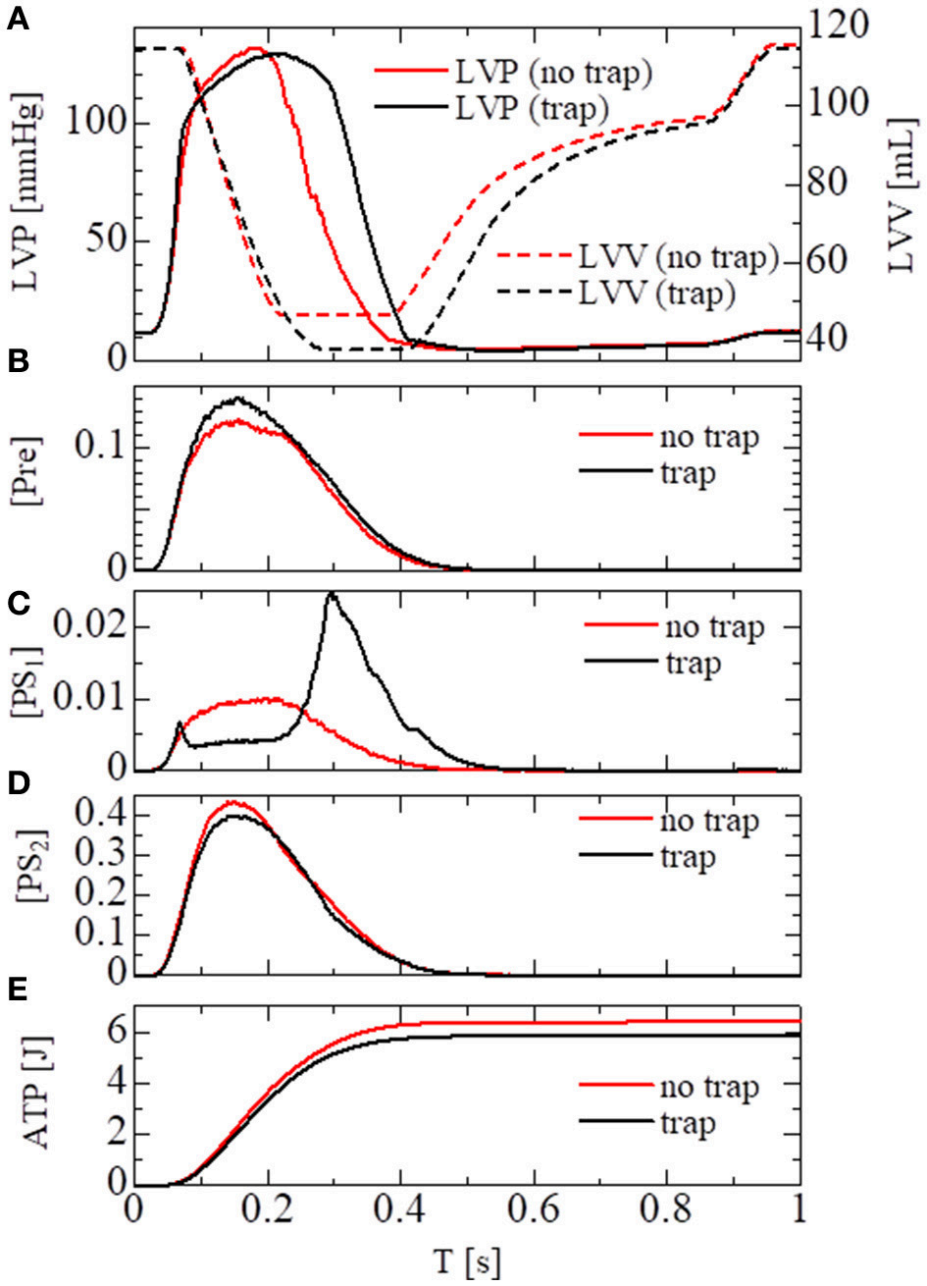
Numerical results of the beating-ventricle simulation using the FEM ventricle model. In each element, the sarcomere model consisting of 8 filament pairs was imbedded. **(A)** The time courses of the left ventricular pressure (solid lines) and volume (broken lines) with the no-trap MH model (red) and the trap model (black). **(B–D)** The time courses of the population ratio of attached MHs in the left ventricular wall classified to the pre-power stroke state (**B**: *Pre*), the first post-power stroke state (**C**: *PS*_1_), and the second post-power stroke state (**D**: *PS*_2_). **(E)** The time courses of the cumulative ATP energy consumption in the left ventricular wall.

In the crossbridge model, the trap and the no-trap models using the various power-stroke free energy potential functions φ_*PS*_ were used, as with the simulations of the single sarcomere oscillation (Table 1). By comparing it with the no-trap model in Figure 11A, the trap mechanism can be seen as contributing to maintaining the high pressure in the last half of the systolic phase. As a result, the blood volume ejected from the left ventricle in the trap model increased to 77 from 68 mL, while the ATP energy consumption of the left ventricular wall decreased to 5.9 from 6.4 J (Figure 11E). This implies that the trap mechanism serves to increase the blood ejection, while also decreasing the energy consumption. Note that the ATP consumption rates were computed by counting the detachments of MHs in *PS*_2_ to those in *N*_*XB*_, which was controlled by the rate constant *D*_*NXB*_ defined in Equation (12).

**Figure 12:**
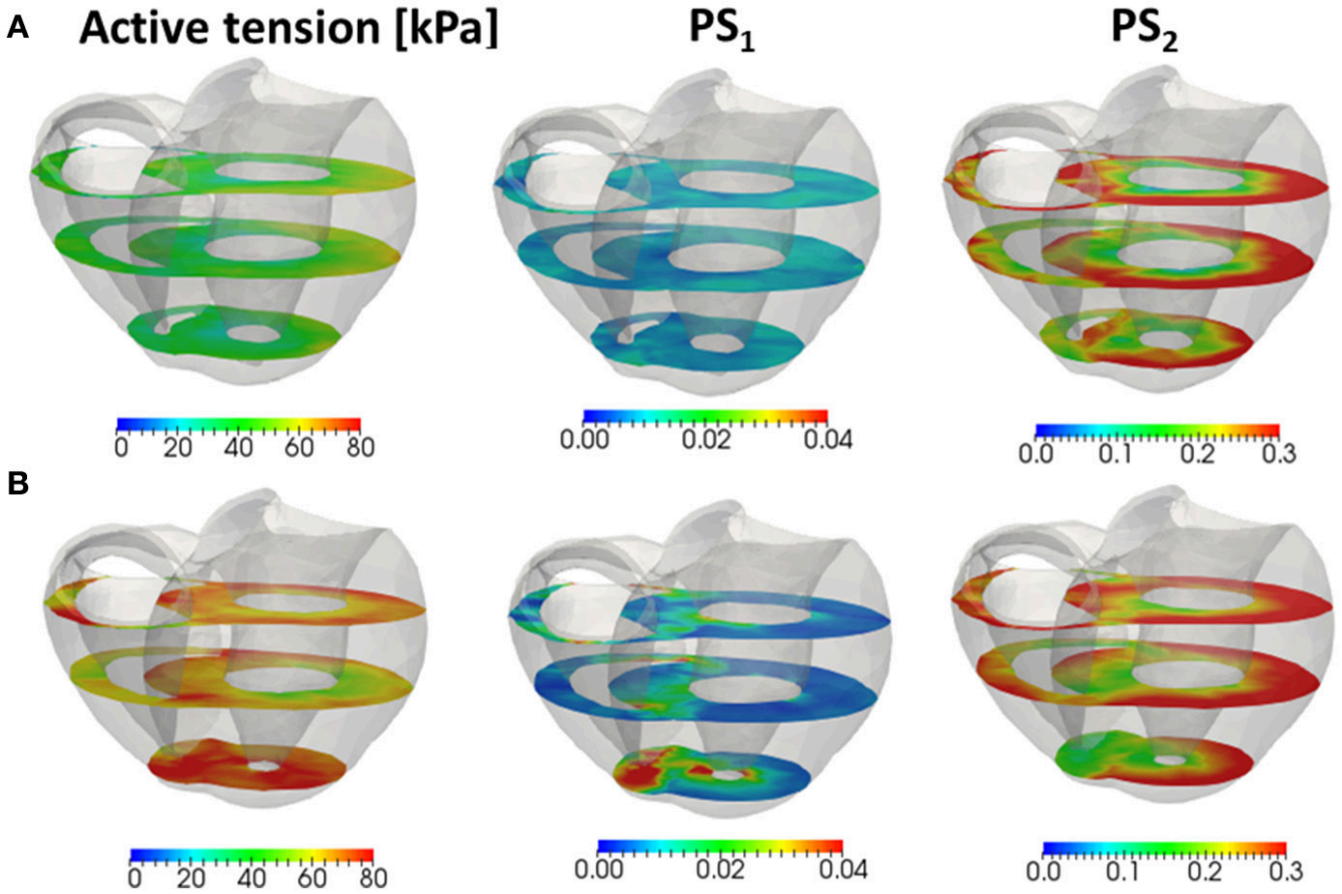
The active tension (left), and the population of MHs in the first post-stroke state *PS*_1_ (center) and the second post-stroke state *PS*_2_ (right) at 0.25 s for the no-trap model **(A)** and the trap model **(B)** in the three cross sections perpendicular to the longitudinal axis.

As shown in Figures 11B–D, two increases in the population of MHs in state *PS*_1_ can be seen; one at the beginning of the systolic phase, and one at the final half. These increases correspond to reversals in the left ventricular pressures of the trap and the no-trap models, as shown in Figure 11A. In the systolic phase, the cardiac myocytes supported their contractile tension along the shared fiber bundle, in which the active stress in Equation (38) provided the great majority of the total stress in Equation (41). Therefore, from the mechanical equilibrium condition along a fiber bundle, the active tensions must be almost equal. If there was a delay in the provision of the active tension, or a relaxation during the intermediate systolic phase at one point of the fiber bundle, this portion quickly became lengthened, and the sarcomeres in the remaining parts shortened until reaching a mechanical equilibrium. Since this transition accompanied decreases in the active tension of the sarcomeres, stopping the process as early as possible was desirable. The trap mechanism could achieve this goal, as shown in Figure 12, in which the distributions of the population of MHs in states *PS*_1_ and *PS*_2_ at the end of the systolic phase (*T* = 0.25 s) were compared. As shown in Figure 12B for the trap model, the higher populations in the *PS*_1_ state were seen in the regions where the populations in state PS_2_ were lower than in the other regions. This indicates that the decrease in the population of MHs at PS_2_ was sufficiently compensated for by the trapped MHs in state *PS*_1_. However, although the population in *PS*_2_ for the no-trap model was similar to one of the trap model, the active tension was nearly half that of the trap model for the entire region (Figure 12A). In particular, the active tensions with the no-trap model were much smaller than those with the trap model, even in the regions with large *PS*_2_ populations. This indicates the importance of maintaining the active tension along the fiber bundle. The distributions of the active tension values and the state populations over the entire cycle are shown in Supplementary Video 1.

**Figure 13:**
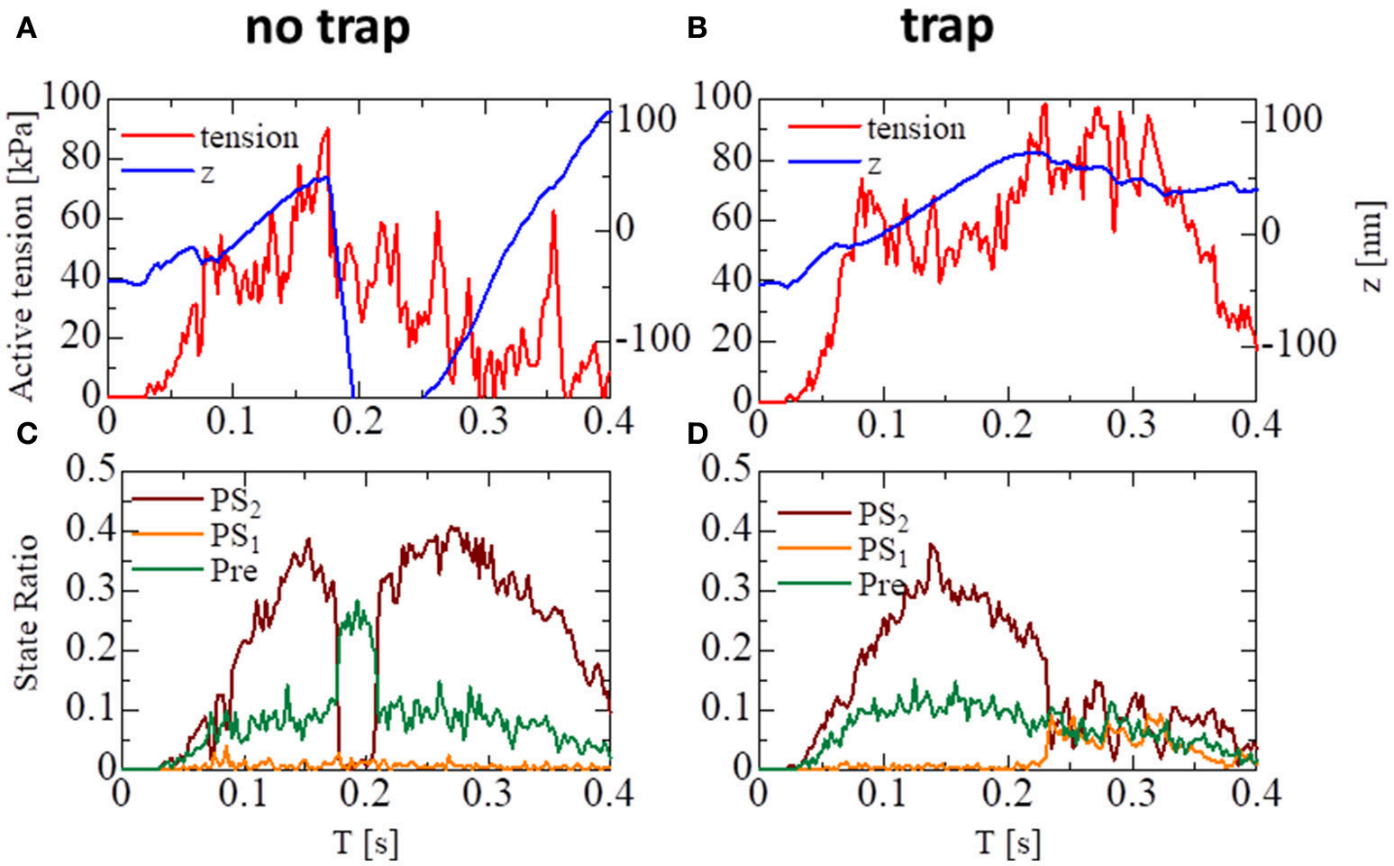
The behavior of the sarcomere in the systolic phase for the no-trap and the trap models imbedded in an identical element at the apical septal segment. **(A,B)** The active tension (red) and the sarcomeric shortening Z (blue). **(C,D)** The population ratio of attached MHs.

The importance of the trap for synchronizing contraction and relaxation over the entire ventricle is further confirmed by Figure 13, in which the behaviors of the sarcomere model with the no-trap and the trap model imbedded with identical elements at the apical septal segment are compared. With the no-trap model (Figure 13A), there was a prominent decline in the sarcomere shortening displacement *z* that accompanied the large drops in the active tension around *T* = 0.18 s. This drop in the active tension was caused by shifts in the population of MHs from *PS*_2_ to the pre-power stroke state *Pre*, as indicated in Figure 13C. As shown previously in the simulations of sarcomere oscillation, each sarcomere had the ability to undergo quick lengthening after a certain duration of contraction. However, the slow decline of LVP in the no-trap model (Figure 11A) at the end of the systolic phase indicates that this characteristic was not necessarily exploited for the quick relaxation of the whole ventricle before the next diastolic phase because the timing of the relaxation changed depending on the Ca^2+^ transients and the sarcomeric movements. Furthermore, a relaxation prior to a sufficient drop in the Ca^2+^-concentration was followed by the next contraction, as shown in Figure 13C, around *T* = 0.2 s. This contraction of the sarcomere did not efficiently contribute to increasing the ejected blood volume, as indicated by LVV in Figure 11A. However, the blood ejection lasted until *T* = 0.3 s in the trap model. Thus, maintaining the active tension with the trapped MHs in *PS*_1_, which corresponded to a rise in the population of *PS*_1_ during the time interval [0.23, 0.3] (Figure 13D), substantially contributed to the ejected blood volume.

**Figure 14:**
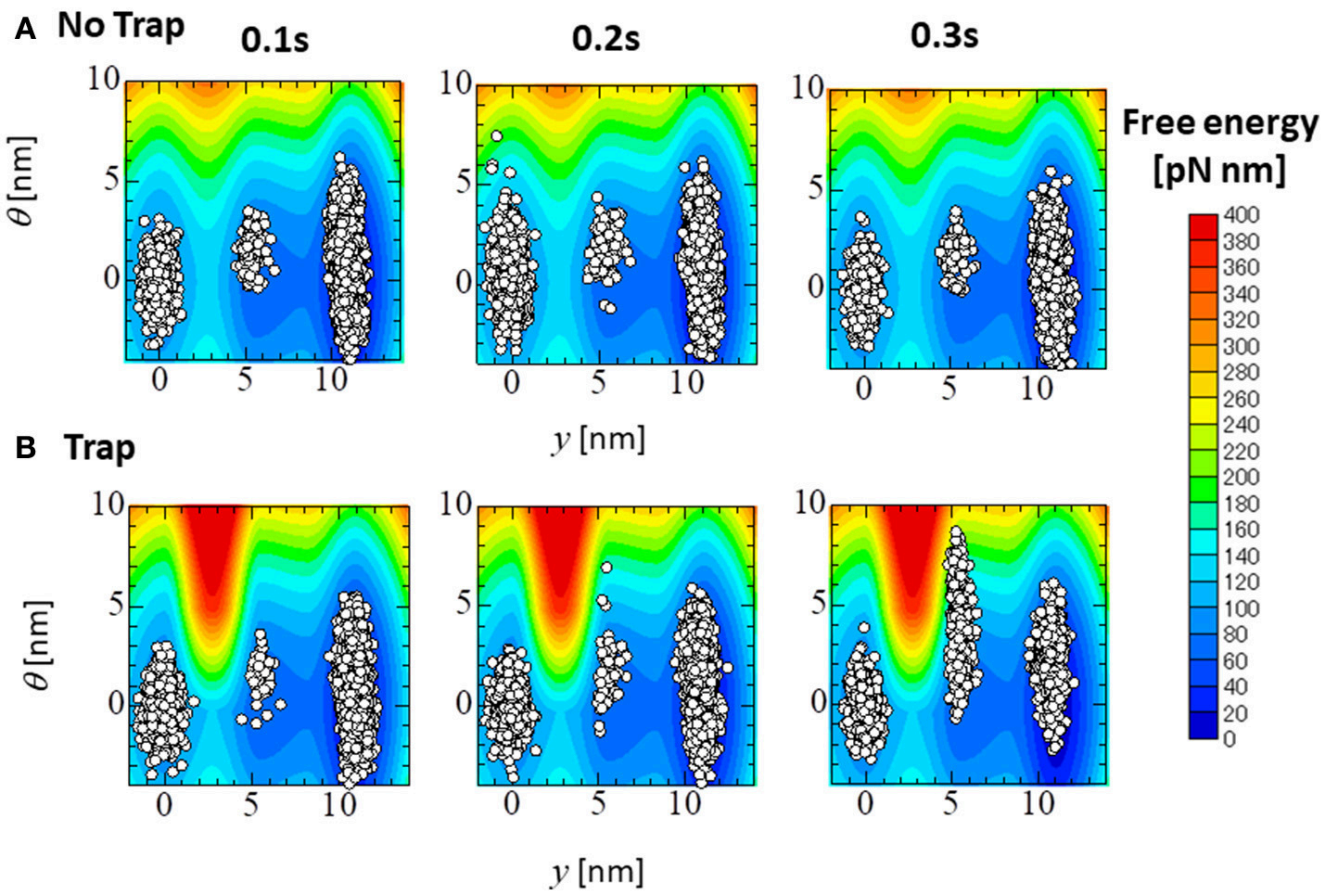
The distribution of the attached MHs at the initial time (*T* = 0.1 s), the peak (*T* = 0.2 s), and the end (*T* = 0.3 s) of the systolic phase for the trap model **(A)** and the no-trap model **(B)** for the (*y*, θ) coordinate. The contours represent the landscape of the free energy φ_*PS*_(θ, *y*) + *W*_*LA*_(θ). The plots are for the attached MHs of the sarcomere models imbedded into the 33 elements in the apical septal segment.

Figure 14 compares the distributions of the attached MHs, which are imbedded in 33 elements at the apical septal segment, in the (*y*, θ) coordinate at *T* = 0.1, 0.2, and 0.3 s of the no-trap and trap models. Although the distributions at the beginning of the systolic phase (*T* = 0.1 s) were nearly the same for both models, differences were found in regions of higher deflection θ in the *Pre* and *PS*_1_ states at the peak of the systolic phase (*T* = 0.2 s), and in *PS*_1_ and *PS*_2_ at the end of the systolic phase (*T* = 0.3 s). Note that the large deflection (θ > 0) of the LA created high strain (ξ > 0) in the rod due to the equilibrium condition for the variable *x* in Equation (1). However, these MHs in the *Pre* state of the no-trap model disappeared quickly due to their large rate of detachment into state *P*_*XB*_ in Equation (11) and Table 1 ($D_{PXB,Pre}=3,000\;{\text{s}}^{-1}$), so that they did not contribute to maintaining the active tension. However, the MHs in state *PS*_1_ were trapped there so long as these myocytes were strongly pulled by the surrounding activated myocytes.

Finally, the computational load and the parallel efficiency were examined. For the microscale computations, the elements of the finite element model were equally distributed to the available cores. But, for the macroscale finite element computations, only one node consisting of 16 cores was used, and the remaining nodes were in the waiting state, since the finite element model was relatively small (7,700 elements). Thus, the parallel efficiency came from the proportion of the macroscale computational time, compared with the total computation time. With the original setup (*n* = 20, 000, *n*_*DA*_ = 10, *n*_*D*_ = 5, Δ*t* = 0.25 ns), the parallel computation with 1,920 cores required 105 h per heartbeat. Within this total elapsed time, 16% was occupied by the macroscale computations. Thus, good parallel efficiency was achieved. Further evaluations of the parallel efficiency are given in the Supplementary Material S7.

## Discussion

### Accuracy, stability, and efficiency of the MTS scheme

The MTS scheme coupled the integration of the molecular variables that use the small time step Δ*t* with the integration of the sarcomere shortening variable *z* that used the coarse time step Δ*T*, which is a large integer multiple of Δ*t*. Since sarcomere shortening is linked to the shortening of the continuum along the fiber orientation by Equation (35), the same coupling scheme can be applied to the coupling with the finite element model. The key point of the proposed MTS scheme is that the active tension at time *T* + Δ*T* is implicitly determined by combining the stretch rate of the continuum along the fiber orientation at *T* + Δ*T*, as given in Equation (37), in which the stiffness of the attached myosin rods during the time interval [*T* : *T* + Δ*T*] given by Equation (29) is used. By applying this implicit scheme, an appropriate time step interval Δ*T* can be chosen for the macroscale computation to diminish the synchronization and communication overhead in the distributed memory parallel system. The accuracy of the MTS scheme, in which the time step ratio was set to 0.25 ns: 5 μs, was validated using a simulation of the spontaneous oscillation of a single sarcomere, and by comparing the numerical results with those computed using equal time intervals.

### Required computational power for the coupled simulation

For the beating-ventricle simulation of ventricle model consisting of 7,600 elements, 105 h were required for each beat using 1,920 cores and a 0.25-ns time step integration in the molecular computations, and a 5-μs time step integration for the macroscopic finite element computation. Within this computation, 84% of the total time was consumed by the microscopic molecular computation. In this simulation, 4 elements were assigned to each core, in which the sarcomere model consisted of 8 filament pairs imbedded in each element. Therefore, the CPU time per filament pair was ~2.8 h. This is the fastest case, not counting the macroscale computational case in which one core was assigned to each filament. Even for the rather coarse mesh model consisting of 7,600 elements, this fastest computation required 60,800 (= 7, 600 × 8) cores. This shows that our application still required huge computational power.

### Potential of the coupled approach

In this paper, an effective utilization of the coupled approach to explore the macroscopic effects of a molecular mechanism was shown. Regarding the molecular mechanism, the power-stroke free energy potential was constructed so as to reproduce the stretch-activation for the single-sarcomere model. In this model, the energy barrier between the pre-power stroke state and the state after the first power stroke was made higher for large positive lever arm deflections, which meant that large loads were imposed on the myosin rods and heads. If the pre-power stroke state and the state after the first power stroke correspond to, respectively, the so-called “P_i_-release state” and “ADP state,” the forward and reversal power stroke transitions accompany the release and the rebinding of inorganic phosphate (P_i_), respectively (Llinas et al., 2015). Thus, if the larger load on the MH closes the channel in which P_i_ travels during the transitions, the height of the free energy barrier could increase. In the proposed numerical model, this hypothesis was reflected by the landscape of the free energy φ_*PS*_(θ, *y*), as mentioned above. The coupled approach revealed that the proposed mechanism for the myosin molecule contributed to maintain the high systolic blood pressure for the appropriate period by synchronizing relaxations along the fiber bundles. Stelzer et al. (2006) discussed the possibility of stretch-activation reinforcing regions where stronger contractile tensions were required during the entire systolic phase, while our numerical results suggest that its function is to reinforce the regions that start relaxation earlier than other regions. Of course, this is still just a hypothesis linking the stretch-activation to the performance of the beating heart. However, this function of stretch-activation function at the end of the systolic phase has gone unnoticed until now.

### Limitations

In the coupling approach, a single half-sarcomere model was directly imbedded into each element of the macroscopic ventricular mesh. This means that the periodically repeated pattern of single sarcomere movement was imposed along the filament direction within each element. Thus, the synchronization of the sarcomeres within each element can be assumed. In reality, relaxations of sarcomeres within the same myofibril are not necessarily synchronized. Thus, even though each of the sarcomeres was stretched quickly during relaxation, as shown in the spontaneous oscillation simulation, the stretch speed of the entire cardiac cell may be slowed due to time lags. One way to account for such an effect in the simulation model is to imbed a myofibril model, in which an adequate number of sarcomeres are connected in series, into each element. Obviously, such an approach requires even greater computational resources.

### New insights of cardiac muscle relaxation in a beating heart

Using the numerical experiments on the single-sarcomere model, spontaneous oscillatory behavior was recovered via the Langevin dynamics model with a simple power-stroke free energy, as in Equation (8) with a constant energy barrier [*E*_*b*1_(θ) ≡ *E*_*b*01_]. The prominent characteristic of this oscillation is the quick lengthening induced by collective reversal strokes (Figure 7A). At first glance, it appears that this mechanism operated by quickly relaxing the muscle against the slow decline of the Ca^2+^ concentration (Inset in Figure 5). However the timing of the lengthening events differ from those in the ventricle wall due to the various feedback signals from the local muscle movements, resulting in the slow decline of the LVP (Figure 11A). Using the numerical experiments on the ventricular model, we see that the trap mechanism contributes to the synchronization of muscle relaxation by halting sarcomeric lengthening if it occurs earlier than in the neighboring muscle. We also see that the same trap mechanism causes the stretch-activation phenomenon at the tissue level.

## Author contributions

TW and TH: designed the project; TW and RK: designed and conceived the numerical model; TW and J-IO: constructed the simulation code and the input data; TW and SS: wrote the paper with input from TH.

### Conflict of interest statement

The authors declare that the research was conducted in the absence of any commercial or financial relationships that could be construed as a potential conflict of interest.
