# Supplementary material for: Coupling Langevin Dynamics With Continuum Mechanics: Exposing the Role of Sarcomere Stretch Activation Mechanisms to Cardiac Function
Source: Front Physiol. 2018 Apr 6;9:333. doi: 10.3389/fphys.2018.00333 (PMC5898180; doi:10.3389/fphys.2018.00333)
Supplement: Supplementary file 2 [file Presentation1.pdf]

# *Supplementary Material*

# Coupling Langevin Dynamics with Continuum Mechanics: Exposing the Role of Sarcomere Stretch Activation Mechanisms to Cardiac Function

**Takumi Washio[1,2], Seiryo Sugiura[1,2], Ryo Kanda[3], Jun-ichi Okada[1,2], and Toshiaki Hisada[1,2]**

[1] UT-Heart Inc., UT Kashiwanoha Campus Satellite, 178-4-4 Wakashiba, Kashiwa, Chiba 277-0871, Japan

[2] Graduate School of Frontier Sciences, University of Tokyo, UT Kashiwanoha Campus Satellite, 178-4-4 Wakashiba, Kashiwa, Chiba 277-0871, Japan

[3] Predictive Health Team, Integrated Research Group, Compass to Healthy Life Research Complex Program, RIKEN, 6-7-1 Minatojima Minamimachi, Chuo-ku, Kobe, Hyogo 650-0047, Japan

*** Correspondence:** Takumi Washio: washio@sml.k.u-tokyo.ac.jp

## S1. Details of the Half-Sarcomere Model

### S1.1 Cooperative Mechanism during Transitions between the Nonbinding State $N_{XB}$ and the Weakly-binding State $P_{XB}$

All parameters introduced in this section are given in Table S1.1. We used the same values as in our previous work (Washio et al., 2016), with the exception of the length of a thin filament, which was set to 1.0 μm for better agreement with a recent measurement (Kolb et al., 2016). In our half-sarcomere model, $n_M$ myosin molecules are arranged on a thick filament at regular intervals, except for the bare zone (B-zone). The thick filament is divided into $n_T$ segments called troponin/tropomyosin (T/T) units (Figure S1.1). Three states, called Ca-off, Ca-on*, and Ca-on, can be assumed by each T/T unit. The transitions between the states of a T/T unit are affected by the $Ca^{2+}$ concentration [Ca] and the states of the MHs below that T/T unit. The corresponding T/T unit index $ia$ of the $i$-th MH in the $j$-th filament is given by:

$$ia = \mathrm{int}\left(\frac{z + 0.5LB + (i - 0.5)S_M + \xi_{i,j} - x_{i,j} - (0.5SL_0 - LA)}{S_T}\right) \qquad (S1.1)$$

Here, $LB$ and $LA$ are the lengths of the B-zone and the thin filament, respectively. $S_M = 0.5(LM - LB)/n_M$ is the spacing of the MHs, and $S_T = LA/n_T$ is the spacing of the T/T units. Note that the corresponding T/T unit exists only if $1 \le ia \le n_T$.

The transition rates between the three states of the T/T unit are defined below. These transition rates are denoted by constants with a prime if there is an MH in a weakly or strongly bound state below the T/T unit, so that Ca binding can be facilitated by nearby cross-bridges.

(1) Ca-off → Ca-on*

$$\bar{K}_{\text{on}}^{*}[\text{Ca}] = \begin{cases} K'^{*}_{\text{on}}[\text{Ca}] & \text{if there is a bound MH below,} \\ K^{*}_{\text{on}}[\text{Ca}] & \text{otherwise.} \end{cases} \quad \text{(S1.2)}$$

(2) Ca-off ← Ca-on*

$$\bar{K}_{\text{off}}^{*} = \begin{cases} K'^{*}_{\text{off}} & \text{if there is a bound MH below,} \\ K^{*}_{\text{off}} & \text{otherwise.} \end{cases} \quad \text{(S1.3)}$$

(3) Ca-on* → Ca-on

$$\bar{K}_{\text{on}}[\text{Ca}] = \begin{cases} K'_{\text{on}}[\text{Ca}] & \text{if there is a bound MH below,} \\ K_{\text{on}}[\text{Ca}] & \text{otherwise.} \end{cases} \quad \text{(S1.4)}$$

(4) Ca-on* ← Ca-on

$$\bar{K}_{\text{off}} = \begin{cases} K'_{\text{off}} & \text{if there is a bound MH below,} \\ K_{\text{off}} & \text{otherwise.} \end{cases} \quad \text{(S1.5)}$$

The transitions between the $N_{XB}$ and $P_{XB}$ states are affected by the status of the T/T unit above it through modifications of $K_{\text{np}}$ and $K_{\text{pn}}$ , as well as by the state of the neighboring MHs through the integer $ng$, where $ng$ $(= 0, 1, \text{or } 2)$ is the number of neighboring MHs in a weakly or strongly bound state.

(1) NXB → PXB

$$R_{NXB\rightarrow PXB} = K_{\text{np}}\gamma^{ng} \quad \text{(S1.6)}$$

(2) NXB ← PXB

$$R_{PXB\rightarrow NXB} = K_{\text{pn}}\gamma^{-ng} \quad \text{(S1.7)}$$

The former is given by:

$$K_{\text{np}} = \begin{cases} \delta_{OV}K_{\text{np1}} & \text{if the T/T unit above is in the Ca-on state,} \\ \delta_{OV}K_{\text{np0}} & \text{otherwise.} \end{cases} \quad \text{(S1.8)}$$

$$K_{\text{pn}} = \begin{cases} K_{\text{pn1}} & \text{if the T/T unit above is in the Ca-on state,} \\ K_{\text{pn0}} & \text{otherwise.} \end{cases} \quad \text{(S1.9)}$$

Here, $\delta_{OV} = 1$ if the MH is located at the single overlap region with the thin filament, otherwise $\delta_{OV} = 0$. This, along with $\gamma^{ng}$ or $\gamma^{-ng}$ $(\gamma = 40)$, represents the nearest-neighbor cooperativity of the MHs, following Rice (2003), which plays an important role for the force-pCa relationship, as shown in S2. We assume that one thin filament in the three-dimensional arrangement corresponds to two thin filaments in our half-sarcomere model. This is because we are assuming that cooperative behavior exists along the tropomyosin and tropomyosin molecules wrapped around the thin filament in a double spiral fashion, and one of the spirals is considered in our half-sarcomere model. The constants $K_{\text{np0}}$, $K_{\text{np1}}$, $K_{\text{pn0}}$, and $K_{\text{pn1}}$ are determined from $Q$, $K_{\text{basic}}$, and $\mu$, as follows:

$$K_{\mathrm{np0}} = \frac{QK_{\mathrm{basic}}}{\mu},\ K_{\mathrm{np1}} = QK_{\mathrm{basic}},\ K_{\mathrm{pn0}} = K_{\mathrm{pn1}} = K_{\mathrm{basic}}\gamma^2 \tag{S1.10}$$

Here, $\mu > 1$ controls the degree of cross-bridge inhibition for T/T units in states other than Ca-on, and $Q$ controls the ratio of binding states of the MHs. The greater the value of $Q$, the larger the ratio of binding states for a given $Ca^{2+}$ concentration. To reproduce the sarcomere length ($SL$) dependence in the contraction force, $Q$ is given as a function of $SL$ by

$$Q(SL) = \begin{cases} Q_0 & , SL \geq SL_Q, \\ Q_0 - \alpha_Q(SL_Q - SL), & SL < SL_Q. \end{cases} \tag{S1.11}$$

**Table S1.1** Parameters for the transitions between $N_{XB}$ and $P_{XB}$, and the sarcomere geometry.

| Parameter | Value | Unit | Parameter | Value | Unit |
|---|---|---|---|---|---|
| **Transition of T/T unit** | | | **Number of MHs and T/T units** | | |
| $K_{\mathrm{on}}^*$ | 150 | $\mu M^{-1}s^{-1}$ | $n_M$ | 38 | Unitless |
| $K_{\mathrm{off}}^*$ | 80 | $s^{-1}$ | $n_T$ | 32 | Unitless |
| $K'^*_{\mathrm{on}}$ | 150 | $\mu M^{-1}s^{-1}$ | **Sarcomere Geometry** | | |
| $K'^*_{\mathrm{off}}$ | 20 | $s^{-1}$ | $LM$ | 1.65 | μm |
| $K_{\mathrm{on}}$ | 150 | $\mu M^{-1}s^{-1}$ | $LB$ | 0.16 | μm |
| $K_{\mathrm{off}}$ | 80 | $s^{-1}$ | $LA$ | 1.0 | μm |
| $K'_{\mathrm{on}}$ | 150 | $\mu M^{-1}s^{-1}$ | $SL_0$ | 1.9 | μm |
| $K'_{\mathrm{off}}$ | 40 | $s^{-1}$ | | | |
| **Transition between $N_{XB}$ and $P_{XB}$** | | | | | |
| $K_{basic}$ | 20.0 | $s^{-1}$ | | | |
| $Q_0$ | 2.2 | Unitless | | | |
| $SL_Q$ | 2.2 | μm | | | |
| $\alpha_Q$ | 1.0 | $\mu m^{-1}$ | | | |
| $\mu$ | 15.0 | Unitless | | | |
| $\gamma$ | 40 | Unitless | | | |

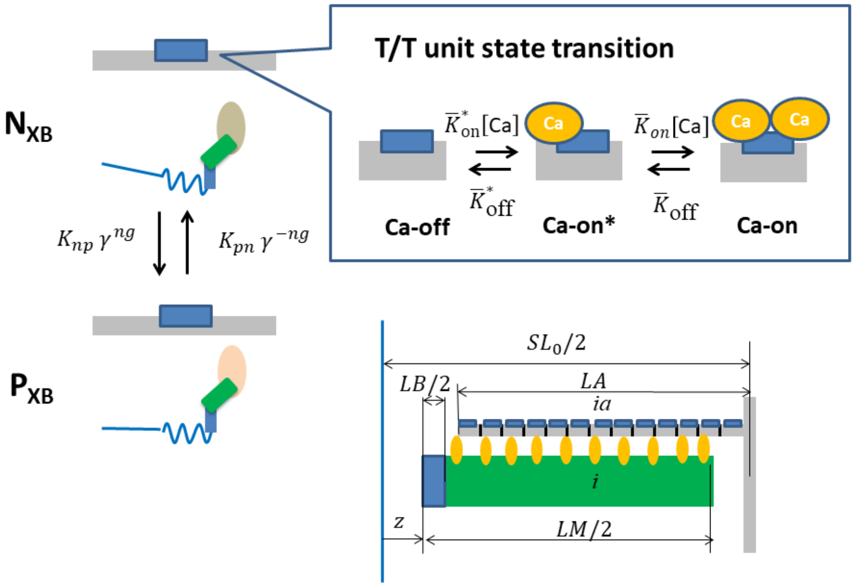


**Figure S1.1** Transitions of the MH states (left), transitions of the T/T unit states (right), and geometry of the half-sarcomere model (right-bottom).

## S1.2 Nonlinear rod strain energy model

The rod strain energy was nonlinear with the generated force, following Kaya (2010). We assume that the myosin rod behaves as a linear spring for positive stretches, whereas nonlinear behavior is introduced for negative stretches, because of the slack region along the myosin rod (Kaya and Higuchi, 2010), as depicted in Figure S1.2. The strain energy $W_{rod}$ is given by integrating the force $F_{rod}$ from $\xi = 0$ given by

$$F_{rod}(\xi) = \begin{cases} b_{xb}k_{xb}(\xi + \xi_1) - F_1, & \xi < -\xi_1 , \\ \dfrac{k_{xb}}{a_{xb}}(\exp(a_{xb}\xi) - 1), & -\xi_1 \leq \xi < 0 , \\ k_{xb}\xi, & \xi \geq 0, \end{cases} \tag{S1.12}$$

where $a_{xb}$ and $F_1$ are determined from the other parameters so that the function $F_{rod}$ and its first derivative are continuous at $\xi = 0$ and $\xi = -\xi_1$:

$$\begin{cases} a_{xb} = -\dfrac{(\ln b_{xb})}{\xi_1}, \\ F_1 = \dfrac{k_{xb}(1 - \exp(-a_{xb}\xi_1))}{a_{xb}}. \end{cases} \tag{S1.13}$$

The parameters adopted in our model are $k_{xb} = 2.8$ pN/nm, $\xi_1$=4.35 nm, and $b_{xb} = 0.05$. The profiles of the resultant force and potential functions are depicted in Figure S1.2.

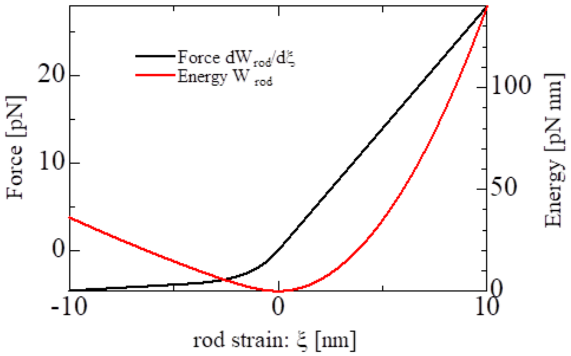


**Figure S1.2** Force $F_{rod}$ and the potential energy $W_{rod}$ for the myosin rod strain $\xi$.

## References for S1


Rice, J. J., Stolovitzky, G., Tu T, and de Tombe, P. P. (2003). Ising model of cardiac thin filament activation with nearest-neighbor cooperative interactions, Biophys J. 84, 897–909. doi: 10.1016/S0006-3495(03)74907-8

Kaya, M, and Higuchi, H. (2010). Non-linear elasticity and an 8 nm working stroke of single myosin molecules in myofilaments, 329, 686–689. doi:10.1126/science.1191484


## S2. Verification of the Basic Properties of the Half-Sarcomere Model

The basic properties of the half-sarcomere model were verified as follows. For the Langevin dynamics, the trap model was applied. All the simulations were executed with the MTS scheme ($\Delta t$ = 0.25 ns, $\Delta T = 5000$ ns:except for S2.4 where $\Delta T = 25$ns was used). The contractile force per one thin filament, $^{T,\Delta T}F$ , in Equation (25) of the main text was averaged over 48 thin filaments ($n_F = 48$). Note that the contractile force computed in the numerical experiments corresponds to the force for one of the double spirals along the thin filament. Therefore, the factor $2/SA_0$ ($SA_0 = 0.001\mu\mathrm{m}^2$, the cross-sectional area of a single thin filament) should be multiplied if one evaluates the tension within the sarcomere. All the numerical simulations were performed with the same parameters as those in the main text, which attempted to reproduce the muscle contraction at normal body temperature, while most of existing experimental data were obtained at lower temperatures. Therefore, we avoid quantitative comparisons with the existing experimental results.

### S2.1 SL and $Ca^{2+}$ Force Relationships

To obtain the force in each case, the simulations were started in the relaxed state (all MHs were in $N_{XB}$) at $T = 0$ s with a given $Ca^{2+}$ concentration [Ca], and the contractile force was averaged over the time interval [0.75 s, 1 s]. In Figure S2.1, the SL and $Ca^{2+}$ dependences of the contractile force are shown. Similar dependences as seen in the experimental results of Kentish et al. (1986) were reproduced. For the SL-force relation, the curvature of the profiles changed from negative to positive as the $Ca^{2+}$ concentration decreased. The $Ca^{2+}$-force relation clearly demonstrated increased $Ca^{2+}$-

sensitivity (a leftward shift of [$Ca_{50}$]) at longer SLs. The Hill coefficient decreased for short values of SL (= 1.7 μm).

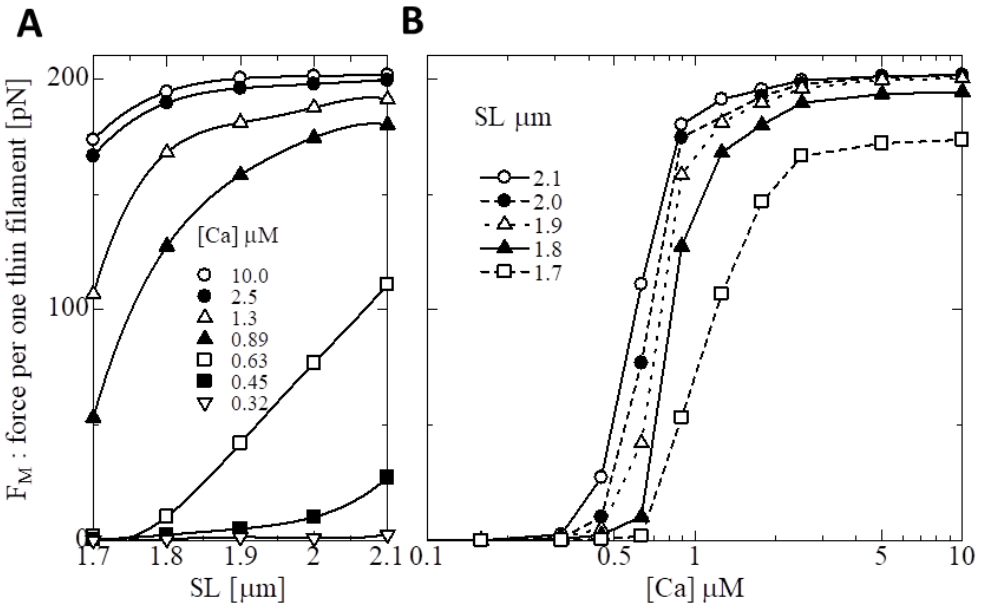


**Figure S2.1** Relationships between force and sarcomere length (A) or [Ca] (B) in the trap model.

## S2.2 Isometric Twitch

A series of isometric twitches were simulated for the $Ca^{2+}$ transient generated by the mid-myocardial cell model proposed by ten Tusscher et al. (2006) while varying the SL (Figure S2.2). As observed by Janssen et al. (1995), the 50% relaxation level was prolonged with increased SL and force, whereas the time to reach the 90% rise level was only slightly prolonged.

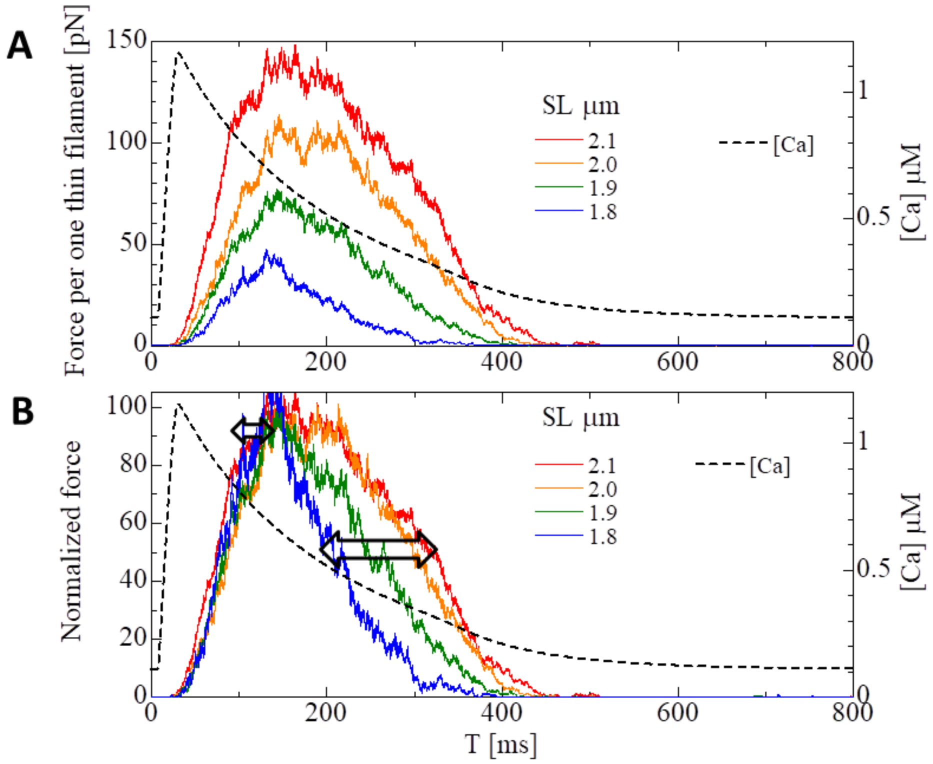


**Figure S2.2** SL dependence of twitch duration. The force of the isometric twitches were compared while varying the SL (A). The same data with the forces normalized by the peak values (B). The applied $Ca^{2+}$ transient is shown by the broken lines.

### S2.3 Force-Velocity Relation

To predict the dependence of the force on the shortening velocity in the trap model, the half-sarcomere excited with a constant [Ca] was quickly released from the initial SL value of 2.3µm with various constant loads (Figure S2.3(A)). Under this isotonic condition, the shortening velocities were measured (Figure S2.3(B)). Physiologically reasonable curves similar to the experimental results given by Piazzesi et al. (1995) were reproduced, with the maximal half-sarcomere shortening velocity close to 3 μm/s.

The SL transient shown in Figure S2.4(A) is similar to the experimental results obtained by Reconditi et al. (2004). As can be seen on the left side of Figure S2.4(B), the initial plateau region $0-5$ ms of the SL transient corresponds to the duration shifting towards the new equilibrium of the isotonic contraction after the non-equilibrium state was induced by the quick release, where a significant number of MHs in the pre-power stroke state (Pre) and the post-first power stroke state ($PS_1$) were transferred rapidly to the post-second power stroke state ($PS_2$). These rapid transitions were induced by a rapid decrease in the rod strain $\xi$ in the Pre and $PS_1$ states, as shown in Figure S2.4(C). In the case of a smaller load (50 pN/Filament), the averaged $\xi$ in $PS_2$ decreases until the increase in the shortening velocity stops. Although the averaged $\xi$ in $PS_2$ is negative after 10 ms, the half-sarcomere produces a positive contractile force equal to 50 pN/Filament. This is due to the nonlinearity of the spring force (Figure S1.2).

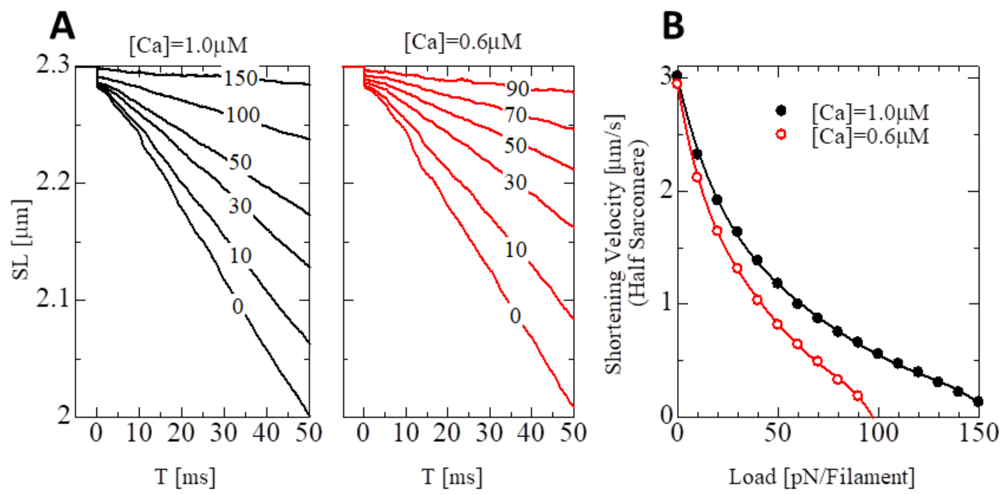


**Figure S2.3** Transients of SLs during the isotonic contractions for constant [Ca]= 1 μM or 0.6 μM with various loads (A). The given loads are represented by the forces [pN] per thin filament. The force-velocity relations were derived by the isotonic contraction experiments (B). For each isotonic case, the shortening velocity was computed from the difference in the SLs at 10 ms and 50 ms after the isotonic contraction was initiated.

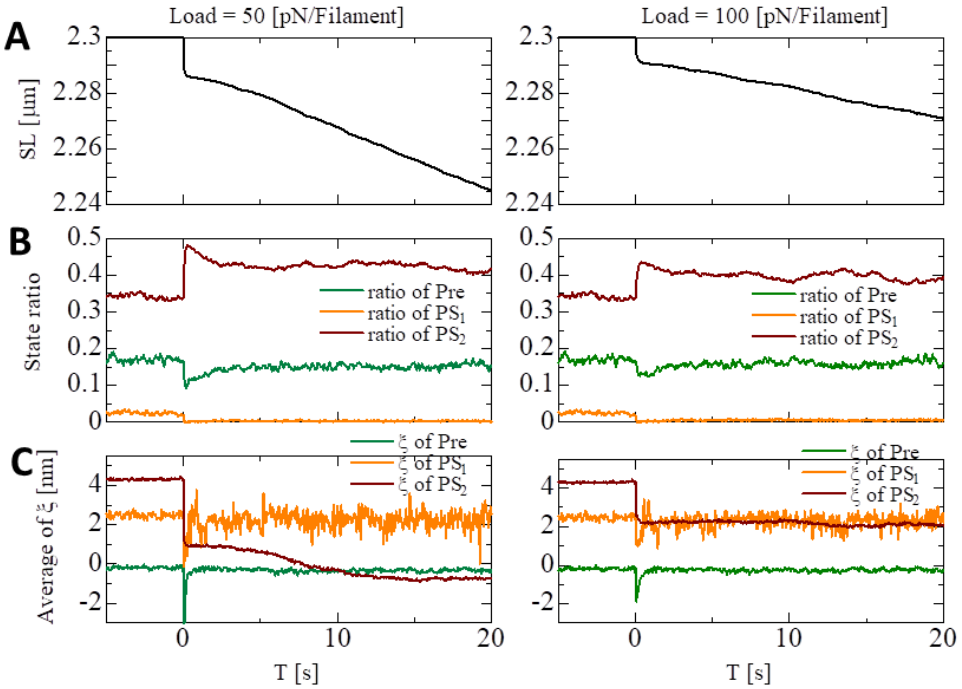


**Figure S2.4** Transients of SL (A), the state ratios (B), and the averaged rod strains $\xi$ (C) for the three binding states during the initial phase of the isotonic contraction for a load 50 pN (left) or 100 pN (right) per thin filament with [Ca] = 1μM.

## S2.4 Tension Recovery for the Step Length Change

To examine the tension recovery after quick shortening, the half-sarcomere model was quickly shortened by 3, 5, or 7 nm within 0.1 ms after the contractile force was developped under a constant [Ca] = 10 μM (Figure S2.5(A)). The shortening effect is clearly recognizable as the sudden decrease of averaged rod strain $\xi$ in the pre-power stroke state (Pre) (Figure S2.5(B)). This induced the quick recovery of tension given by the collective power stroke transition from Pre to $PS_2$ (Figures S2.5 C and D ).

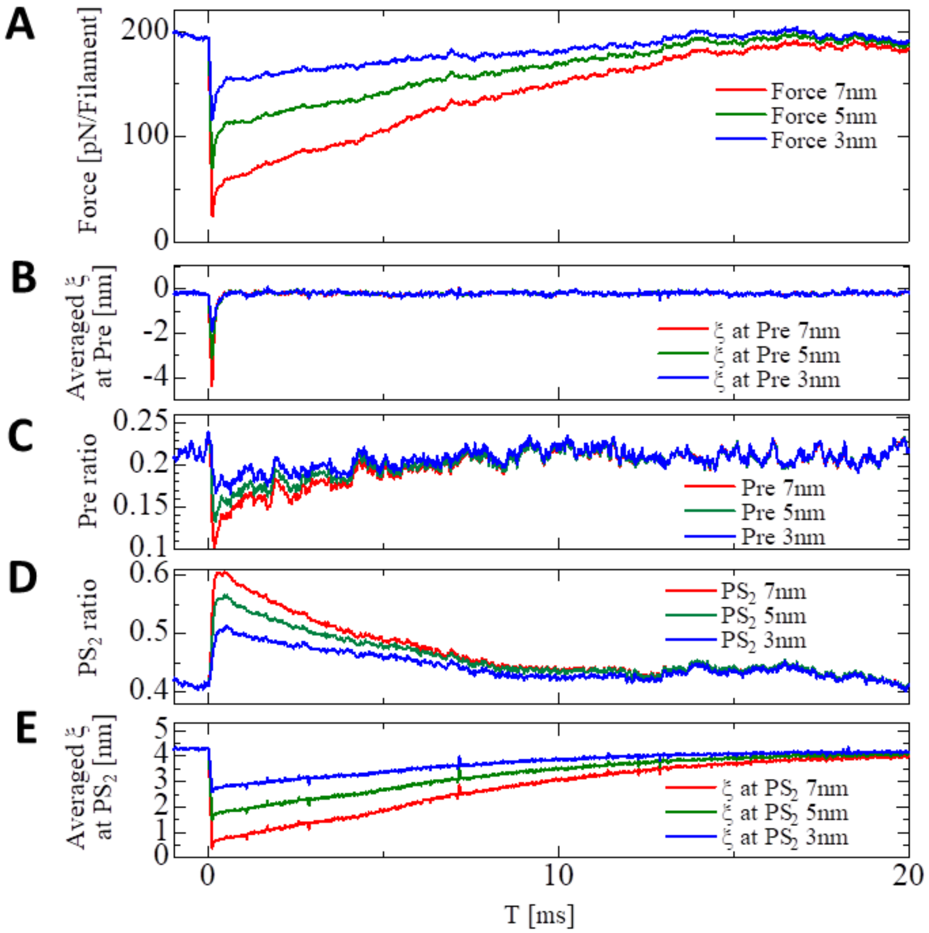


**Figure S2.5** Tension recovery after a quick shortening of 3, 5, or 7 nm of the half-sarcomere (A). The length change was given over [0 ms, 0.1 ms] after the contractile force was developped with [Ca] = 10 μM and SL = 1.9μm. The transients of the state ratios of Pre and $PS_2$ are given in (C) and (D), respectively. The averaged rod strains for Pre and $PS_2$ are given in (B) and (E), respectively.

## References for S2

Kentish, J. C., ter Keurs, H. E., Ricciardi, L., Bucx, J. J., and Noble, M. I. (1986). Comparison between the sarcomere length–force relations of intact and skinned trabeculae from rat right ventricle: influence of calcium concentrations on these relations. Circ. Res. 58, 755–768.

Kolb, J., Li, F., Methawasin, M., Adler, M., Escobar, Y. N., Nedrud, J., Pappas, C. T., Harris, S. P., and Granzier, H. (2016). Thin filament length in the cardiac sarcomere varies with sarcomere length but is independent of titin and nebulin. J Mol Cell Cardiol. 97, 286–294. doi: 10.1016/j.yjmcc.2016.04.013.

ten Tusscher, K. H. W. J., and Panfilov, A. V. (2006). Alternans and spiral breakup in a human ventricular tissue model. Am J Physiol Heart and Circ Physiol. 291. H1088–H1100. doi:717 10.1152/ajpheart.00109.2006

Janssen, P. M., and Hunter, W. C. (1995). Force, not sarcomere length, correlates with prolongation of isosarcometric contraction. Am. J. Physiol. 269, H676–H685. doi: 10.1152/ajpheart.1995.269.2.H676

Piazzesi, G., and Lombardi V. (1995). A cross-bridge model that is able to explain mechanical and energetic properties of shortening muscle. Biophys J. 68, 1966–79. doi: 10.1016/S0006-3495(95)80374-7

Reconditi, M., Linari, M., Lucii, L., Stewart, A., Sun, Y. B., Boesecke, P., Narayanan, T., Fischetti, R. F., Irving, T., Piazzesi, G., Irving, M., Lombardi, V. (2004). The myosin motor in muscle generates a smaller and slower working stroke at higher load. Nature 428, 578–581. doi:10.1038/nature02380

Washio, T., Yoneda, K., Okada, J., Kariya, T., Sugiura, S., and Hisada, T. (2016). Ventricular fiber optimization utilizing the branching structure, I*nt. J. Numer. Method Biomed. Eng.* 32, e02753. doi: 10.1002/cnm.2753

## S3. Derivation of the Active Stress Tensor and its Stiffness

The muscle power $P$ per unit volume delivered by the contractile tension $T_f$ (per unit area in the reference configuration) in the direction specified by the unit vector $\boldsymbol{f}$ (in the reference configuration) is given by the product of $T_f$ and the time derivative of the stretching $\lambda = \left\| \frac{\partial x}{\partial X} \boldsymbol{f} \right\|$ along $\boldsymbol{f}$:

$$P = -T_f \dot{\lambda} = -\frac{T_f}{\lambda} \sum_{i,j,k=1}^{3} \frac{\partial x_i}{\partial X_j} f_j \frac{\partial \dot{x}_i}{\partial X_k} f_k = -\frac{T_f}{\lambda} \left( \frac{\partial \boldsymbol{x}}{\partial \boldsymbol{X}} \cdot \boldsymbol{f} \otimes \boldsymbol{f} \right) : \frac{\partial \dot{\boldsymbol{x}}}{\partial \boldsymbol{X}} \qquad \text{(S3.1)}$$

Here, the : symbol denotes the dot product of the two tensors. Thus, the active stress can be represented by the first Piola-Kirchhoff stress tensor:

$$\boldsymbol{\Pi}_{\mathrm{act}} = \frac{T_f}{\lambda} \boldsymbol{f} \otimes \boldsymbol{f} \cdot \left( \frac{\partial \boldsymbol{x}}{\partial \boldsymbol{X}} \right)^T \qquad \text{(S3.2)}$$

As a result, Equation (S3.1) can be rewritten as

$$P = -\boldsymbol{\Pi}_{\mathrm{act}}{}^{T} : \frac{\partial \dot{\boldsymbol{x}}}{\partial \boldsymbol{X}} \tag{S3.3}$$

The application of the first Piola-Kirchhoff stress tensor $\boldsymbol{\Pi}_{\mathrm{act}}$ to an area element $\boldsymbol{N}dA$ in the reference configuration, in which the normal vector $\boldsymbol{N}$ points outward from the area, yields the traction force $\boldsymbol{dt}$ in the current configuration, as follows:

$$\boldsymbol{dt} = \boldsymbol{\Pi}_{\mathrm{act}}{}^{T}\boldsymbol{N}dA = \frac{T_f}{\lambda}\frac{\partial \boldsymbol{x}}{\partial \boldsymbol{X}}\boldsymbol{f}(\boldsymbol{f}\cdot\boldsymbol{N})dA = T_f\tilde{\boldsymbol{f}}(\boldsymbol{f}\cdot\boldsymbol{N})dA \tag{S3.4}$$

Here, $\tilde{\boldsymbol{f}} = \frac{1}{\lambda}\frac{\partial x}{\partial X}\boldsymbol{f}$ is the unit vector directed along the fiber orientation direction in the current configuration, and $(\boldsymbol{f}\cdot\boldsymbol{N})$ is proportional to the number of actin filaments that pass through the unit area perpendicular to $\boldsymbol{N}$. Thus, Equation (S3.4) indicates that the active stress tensor in Equation (S3.2) is based on the muscle power $P$, which corresponds with the usual stress tensor defined by the traction force.

From Equation (S3.2), the active stress tensor is represented by the second Piola-Kirchhoff stress tensor:

$$\boldsymbol{S}_{\mathrm{act}} = \frac{T_f}{\lambda}\boldsymbol{f}\otimes\boldsymbol{f} \tag{S3.5}$$

To perform an implicit time integration scheme, such as the Newmark-β method, we need to compute the derivative of the stress tensor with respect to the nodal displacement vector $\boldsymbol{u}$, as well as its time derivative $\dot{\boldsymbol{u}}$. In Equation (S3.5), $\lambda$ is a function of $\boldsymbol{u}$, and $T_f$ is a function of $\boldsymbol{u}$ and $\dot{\boldsymbol{u}}$ , because $T_f$ in Equation (38) contains $\dot{\lambda}$ (which is a function of $\boldsymbol{u}$ and $\dot{\boldsymbol{u}}$) :

$$\begin{cases} \lambda = \left(1 + 2\boldsymbol{f}\cdot\left(\frac{\partial \boldsymbol{u}}{\partial \boldsymbol{X}}\boldsymbol{f}\right) + \left(\frac{\partial \boldsymbol{u}}{\partial \boldsymbol{X}}\boldsymbol{f}\right)\cdot\left(\frac{\partial \boldsymbol{u}}{\partial \boldsymbol{X}}\boldsymbol{f}\right)\right)^{1/2} \\ \dot{\lambda} = \frac{1}{\lambda}\left(\boldsymbol{f}\cdot\left(\frac{\partial \dot{\boldsymbol{u}}}{\partial \boldsymbol{X}}\boldsymbol{f}\right) + \left(\frac{\partial \boldsymbol{u}}{\partial \boldsymbol{X}}\boldsymbol{f}\right)\cdot\left(\frac{\partial \dot{\boldsymbol{u}}}{\partial \boldsymbol{X}}\boldsymbol{f}\right)\right) \end{cases} \tag{S3.6}$$

Thus, the derivative of $\boldsymbol{S}_{\mathrm{act}}$ can be computed by

$$\boldsymbol{\delta S}_{\mathrm{act}} = \frac{T_f}{\lambda}\left(-\frac{\delta\lambda}{\lambda} + \frac{dT_f}{d\dot{\lambda}}\delta\dot{\lambda}\right)\boldsymbol{f}\otimes\boldsymbol{f} \tag{S3.7}$$

Now, from Equation (37), the derivative of $T_f$ is given by

$$\frac{dT_f}{d\dot{\lambda}} = \Delta T\frac{R_S SL_0}{SA_0}{}^{T,\Delta T}K_F \tag{S3.8}$$

and the derivatives of $\lambda$ and $\dot{\lambda}$ are, respectively,

$$\delta\lambda = \frac{1}{\lambda}\left(\boldsymbol{f} + \frac{\partial \boldsymbol{u}}{\partial \boldsymbol{X}}\boldsymbol{f}\right) \cdot \left(\frac{\partial \boldsymbol{\delta u}}{\partial \boldsymbol{X}}\boldsymbol{f}\right) \tag{S3.9}$$

and

$$\delta\dot{\lambda} = \frac{1}{\lambda}\left(\frac{\partial \boldsymbol{u}}{\partial \boldsymbol{X}}\boldsymbol{f} - \frac{\dot{\lambda}}{\lambda}\left(\boldsymbol{f} + \frac{\partial \boldsymbol{u}}{\partial \boldsymbol{X}}\boldsymbol{f}\right)\right) \cdot \left(\frac{\partial \boldsymbol{\delta u}}{\partial \boldsymbol{X}}\boldsymbol{f}\right) + \frac{1}{\lambda}\left(\boldsymbol{f} + \frac{\partial \boldsymbol{u}}{\partial \boldsymbol{X}}\boldsymbol{f}\right) \cdot \left(\frac{\partial \boldsymbol{\delta \dot{u}}}{\partial \boldsymbol{X}}\boldsymbol{f}\right) \tag{S3.10}$$

## S4 Passive and Viscous Parts of the Ventricle Model

The passive stress tensor is given by the deformation potential function $W_{pas}$ as

$$\boldsymbol{\Pi}_{\text{pas}} = \frac{\partial W_{\text{pas}}}{\partial \boldsymbol{Z}}^{T} \tag{S4.1}$$

The potential function $W_{pas}$ is determined by a macroscopic passive potential:

$$W_{\text{pas}} = c_1(\tilde{I}_1 - 3) + c_u \frac{\exp(Q_u) - 1}{2} + R_S W_{\text{sar}} \tag{S4.2}$$

where $\tilde{I}_1$ is is the reduced invariant defined as

$$\tilde{I}_1 = \det(\boldsymbol{C})^{-\frac{1}{3}}\text{Tr}(\boldsymbol{C}) \tag{S4.3}$$

with the right Cauchy-Green deformation tensor $\boldsymbol{C} = \boldsymbol{F}^T\boldsymbol{F}$. $Q_u$ is a quadratic form of the Green-Lagrange strain tensor (Usyk et al., 2000):

$$Q_u = b_{ff}E_{ff}^2 + b_{ss}E_{ss}^2 + b_{nn}E_{nn}^2 + 2b_{fs}E_{fs}^2 + 2b_{fn}E_{fn}^2 + 2b_{sn}E_{sn}^2 \tag{S4.4}$$

where the components are defined based on the fiber-sheet structure of the muscle walls as

$$\begin{cases} E_{ff} = \boldsymbol{E}: \boldsymbol{f} \otimes \boldsymbol{f}, E_{ss} = \boldsymbol{E}: \boldsymbol{s} \otimes \boldsymbol{s}, E_{nn} = \boldsymbol{E}: \boldsymbol{n} \otimes \boldsymbol{n} \\ E_{fs} = \boldsymbol{E}: \boldsymbol{f} \otimes \boldsymbol{s}, E_{fn} = \boldsymbol{E}: \boldsymbol{f} \otimes \boldsymbol{n}, E_{sn} = \boldsymbol{E}: \boldsymbol{s} \otimes \boldsymbol{n} \end{cases} \tag{S4.5}$$

Here, $\{\boldsymbol{f}, \boldsymbol{s}, \boldsymbol{n}\}$ is the orthonormal basis of the ventricle walls that determines the fiber and laminar structures. $R_S$ denotes the volume ratio of the sarcomere in the ventricle muscle wall, and $W_{\text{sar}}$ is the potential function introduced by the material properties of the sarcomere mechanics. The thick filaments are connected to the Z-line with another filamentous protein called titin. These proteins are believed to prevent overstretching of the sarcomere, and the presence of the thick filaments is assumed to prevent too much shortening. These effects are modeled by introducing the potential as a function of the stretch $\lambda$:

$$W_{\text{sar}}(\lambda)=\begin{cases}\dfrac{c_{\text{mf}}}{4}(\lambda-\lambda_{\text{mf}})^4, & \lambda<\lambda_{\text{mf}}\\ 0, & \lambda_{\text{mf}}\le\lambda\le\lambda_{\text{titin}}\\ \dfrac{c_{\text{titin}}}{3}(\lambda-\lambda_{\text{titin}})^3, & \lambda>\lambda_{\text{titin}}\end{cases}\quad (S4.6)$$

Here, $\lambda_{\text{mf}}$ is the shortening threshold of $\lambda$ of the thick filament, and $\lambda_{\text{titin}}$ is the elongation threshold of the titin.

For the viscous part, the Newtonian viscosity is given by

$$\boldsymbol{\Pi}_{\text{vis}}=2\mu_S J\boldsymbol{F}^{-1}\boldsymbol{D}_S \quad (S4.7)$$

where $\mu_S$ is the viscosity coefficient, and $\boldsymbol{D}_S$ is the deformation velocity tensor defined as:

$$\boldsymbol{D}_S=\frac{1}{2}\left(\frac{\partial\dot{\boldsymbol{u}}}{\partial\boldsymbol{x}}+\frac{\partial\dot{\boldsymbol{u}}}{\partial\boldsymbol{x}}^T\right) \quad (S4.8)$$

Note that the derivatives are given with respect to the Eulerian coordinates $\boldsymbol{x}$.

Table S4.1 Parameters for the muscle material properties in the biventricular model.

| **Parameter** | **Value** | **Unit** | **Parameter** | **Value** | **Unit** |
|---|---|---|---|---|---|
| **Muscle Passive** | | | **Sarcomere** | | |
| $c_1$ | 71.8 | Pa | $SA_0$ | 0.001 | $\mu m^2$ |
| $c_u$ | 600 | Pa | $R_S$ | 0.5 | Unitless |
| $b_{ff}$ | 5 | Unitless | $c_{\text{mf}}$ | 10 | GPa |
| $b_{ss}$ | 6 | Unitless | $\lambda_{\text{mf}}$ | 0.87 | Unitless |
| $b_{nn}$ | 3 | Unitless | $c_{\text{titin}}$ | 230 | KPa |
| $b_{fs}$ | 10 | Unitless | $\lambda_{\text{titin}}$ | 1.0 | Unitless |
| $b_{fn}$ | 2 | Unitless | | | |
| $b_{sn}$ | 2 | Unitless | | | |
| $\mu_S$ | 36.66 | $\text{Pa}\cdot\text{s}$ | | | |
| $\kappa$ | 200 | KPa | | | |
| $\gamma$ | 40 | Unitless | | | |

**References for S4**

Usyk, T. P., Mazhari, R., and McCulloch, A. D. (2000). Effect of laminar orthotrophic myofiber architecture on regional stress and strain in the canine left ventricle. Journal of Elasticity 61,143–164. doi: https://doi.org/10.1023/A:1010883920374

## S5. Circulatory System

As the atrial model, the formulations by Kaye et al. (2014) were applied. The left and right atrial pressure $P_A$ is related to the time-varying elasticity $e_A$ and the chamber volume $V_A$ as follows. Hereafter, the subscript “A” stands for “LA” or “RA” for the left or the right atrium, respectively.

$$P_A(e_A, V_A) = P_{A,ed}(V_A) + e_A\left(P_{A,es}(V_A) - P_{A,ed}(V_A)\right) \tag{S5.1}$$

Here, the functions $P_{A,ed}$ and $P_{A,es}$ give, respectively, the pressure at the end of the diastolic and systolic phases for chamber volume $V_A$. These functions are defined by

$$\begin{cases} P_{A,ed}(V_A) = \beta_A\left(\exp\left(\alpha_A(V_A - V_{A,0})\right) - 1\right) \\ P_{A,es}(V_A) = E_{A,es}(V_A - V_{A,0}) \end{cases} \tag{S5.2}$$

The time-varying elastance $e_A$ is given as a function of the time $T$ as follows:

$$e_A(T) = \begin{cases} \frac{1}{2}\left(\sin\pi\left(\frac{T-T_0}{T_{\max}} - \frac{1}{2}\right) + 1\right), & T \leq \frac{3}{2}T_{\max} + T_0 \\ \frac{1}{2}\exp\left(-\left(T-T_0 - \frac{3}{2}T_{\max}\right)/\tau_A\right), & T > \frac{3}{2}T_{\max} + T_0 \end{cases} \tag{S5.3}$$

Here, $T_0$ is the start time of atrial excitation, $T_{\max}$ is the time to maximal chamber elastance, and $\tau_A$ is the time constant of relaxation. Together with the other part of the circuit model of Figure 5 in the main text, the overall equations for pulmonary circulation are given by

$$\begin{cases} F_{PA} - H(\bar{F}_{PA})\bar{F}_{PA} = 0 \\ \dot{Q}_{AP} + F_{PA} + \dfrac{1}{R_{AP}}\left(\dfrac{Q_{AP}}{C_{AP}} - \dfrac{Q_{VP}}{C_{VP}}\right) = 0 \\ \dot{Q}_{PV} - \dfrac{1}{R_{AP}}\left(\dfrac{Q_{AP}}{C_{AP}} - \dfrac{Q_{VP}}{C_{VP}}\right) + \dfrac{1}{R_{VP}}\left(\dfrac{Q_{VP}}{C_{VP}} - P_{LA}(e_{LA}, V_{LA})\right) = 0 \\ F_{MI} - H(\bar{F}_{MI})\bar{F}_{MI} = 0 \\ \dot{V}_{LA} - \dfrac{1}{R_{VP}}\left(\dfrac{Q_{VP}}{C_{VP}} - P_{LA}(e_{LA}, V_{LA})\right) - F_{MI} = 0 \end{cases} \tag{S5.4}$$

Here, $C$ denotes the pulmonary venous compliance, and $Q$ denotes the increase of the blood volume from zero pressure. $\bar{F}_{PA}$ and $\bar{F}_{MI}$, the flow rates in the case of no rectification, are given by

$$\begin{cases} \bar{F}_{PA} = \dfrac{1}{R_{PA}}\left(P_R - \dfrac{Q_{AP}}{C_{AP}}\right) \\ \bar{F}_{MI} = \dfrac{1}{R_{LA}}\left(P_{LA}(e_{LA}, V_{LA}) - P_L\right) \end{cases} \tag{S5.5}$$

Similarly, the overall equations for the systemic circulation are given by

$$\begin{cases} F_{AO} - H(\bar{F}_{AO})\bar{F}_{AO} = 0 \\ \dot{Q}_A + F_{AO} + \dfrac{1}{R_A}\left(\dfrac{Q_A}{C_A} - \dfrac{Q_{VS}}{C_{VS}}\right) = 0 \\ \dot{Q}_{VS} - \dfrac{1}{R_A}\left(\dfrac{Q_A}{C_A} - \dfrac{Q_{VS}}{C_{VS}}\right) + \dfrac{1}{R_{VS}}\left(\dfrac{Q_{VS}}{C_{VS}} - P_{RA}(e_{RA}, V_{RA})\right) = 0 \\ F_{TR} - H(\bar{F}_{TR})\bar{F}_{TR} = 0 \\ \dot{V}_{RA} - \dfrac{1}{R_{VS}}\left(\dfrac{Q_{VS}}{C_{VS}} - P_{RA}(e_{RA}, V_{RA})\right) - F_{TR} = 0 \end{cases} \tag{S5.6}$$

with flow rates (for no rectification)

$$\begin{cases} \bar{F}_{AO} = \dfrac{1}{R_C}\left(P_L - \dfrac{Q_A}{C_A}\right) \\ \bar{F}_{TR} = \dfrac{1}{R_{RA}}\left(P_{RA}(e_{RA}, V_{RA}) - P_R\right) \end{cases} \tag{S5.7}$$

The parameters adopted in our simulation are listed in Table S5.1. The parameter values were chosen to reproduce the temporal changes in ventricular blood pressure for a standard healthy heart.

**Table S5.1** Parameters for the pulmonary and systemic circulations, with $T_{\text{cycle}}$ standing for the period of a heartbeat

| **Parameter** | **Value** | **Unit** | **Parameter** | **Value** | **Unit** |
|---|---|---|---|---|---|
| **Pulmonary Circulation** | | | **Systemic Circulation** | | |
| $\boldsymbol{C_{AP}}$ | 3.42 | $\text{ml} \cdot \text{mmHg}^{-1}$ | $C_A$ | 1.64 | $\text{ml} \cdot \text{mmHg}^{-1}$ |
| $\boldsymbol{C_{VP}}$ | 7.52 | $\text{ml} \cdot \text{mmHg}^{-1}$ | $C_{VS}$ | 67.3 | $\text{ml} \cdot \text{mmHg}^{-1}$ |
| $\boldsymbol{R_{PA}}$ | 0.017 | $\text{mmHg} \cdot \text{s} \cdot \text{ml}^{-1}$ | $R_C$ | 0.043 | $\text{mmHg} \cdot \text{s} \cdot \text{ml}^{-1}$ |
| $\boldsymbol{R_{AP}}$ | 0.139 | $\text{mmHg} \cdot \text{s} \cdot \text{ml}^{-1}$ | $R_A$ | 1.18 | $\text{mmHg} \cdot \text{s} \cdot \text{ml}^{-1}$ |
| $\boldsymbol{R_{VP}}$ | 0.019 | $\text{mmHg} \cdot \text{s} \cdot \text{ml}^{-1}$ | $R_{VS}$ | 0.060 | $\text{mmHg} \cdot \text{s} \cdot \text{ml}^{-1}$ |
| $\boldsymbol{R_{LA}}$ | 0.0025 | $\text{mmHg} \cdot \text{s} \cdot \text{ml}^{-1}$ | $R_{RA}$ | 0.0025 | $\text{mmHg} \cdot \text{s} \cdot \text{ml}^{-1}$ |
| Left Atrium | | | **Right Atrium** | | |

| | | | | | |
|---|---|---|---|---|---|
| $\boldsymbol{\alpha_{LA}}$ | 0.044 | $\mathrm{ml}^{-1}$ | $\alpha_{RA}$ | 0.044 | $\mathrm{ml}^{-1}$ |
| $\boldsymbol{\beta_{LA}}$ | 0.3 | mmHg | $\beta_{RA}$ | 0.3 | mmHg |
| $\boldsymbol{E_{LA,es}}$ | 0.3 | $\mathrm{mmHg}\cdot\mathrm{ml}^{-1}$ | $E_{RA,es}$ | 0.25 | $\mathrm{mmHg}\cdot\mathrm{ml}^{-1}$ |
| $\boldsymbol{V_{LA,0}}$ | 5.0 | ml | $V_{RA,0}$ | 5.0 | ml |
| $\boldsymbol{\tau_{LA}}$ | 0.025 | s | $\tau_{RA}$ | 0.025 | s |
| $\boldsymbol{T_{\max}}$ | 0.125 | s | $T_{\max}$ | 0.125 | s |
| $\boldsymbol{T_{\mathrm{cycle}}}$ | 0.15 | s | $T_{\mathrm{cycle}} - T_0$ | 0.15 | s |

## References for S5

Kaye, D., Shah, S. J., Borlaug, B. A., Gustafsson, F., Komtebedde, J., Kubo, S., Magnin, C., Maurer, M. S., Feldman, T., and Burkhoff, D. (2014). Effects of an interatrial shunt on rest and exercise hemodynamics: results of a computer simulation in heart failure. J Card Fail. 20, 212–221. doi: 10.1016/j.cardfail.2014.01.005.

## S6. Newmark-beta Time Integration for the Macroscopic Nonlinear Equation

In our process, the combined system composed of the mechanical equations for the biventricular model and the blood circulation equations were simultaneously solved implicitly using the Newmark-beta scheme. The combined system of equations is given by the following three formulas, for the biventricular FEM, pulmonary circulation, and systemic circulation models.

$$\begin{cases} \displaystyle\int_{\Omega} \boldsymbol{\delta\dot{u}} \cdot \rho \ddot{\boldsymbol{u}}\, d\Omega + \int_{\Omega} \boldsymbol{\delta\dot{Z}} : (\boldsymbol{\Pi} + 2pJ\boldsymbol{F}^{-1})^{T}\, d\Omega \\ \qquad \displaystyle -P_L \int_{\Gamma_L} \boldsymbol{\delta\dot{u}} \cdot \boldsymbol{n}\, d\Gamma_L - P_R \int_{\Gamma_L} \boldsymbol{\delta\dot{u}} \cdot \boldsymbol{n}\, d\Gamma_R = 0 \\ \displaystyle\int_{\Omega} \delta p \left( 2(J-1) - \frac{p}{\kappa} \right) d\Omega = 0 \\ \displaystyle\delta P_L \left( \int_{\Gamma_L} \dot{\boldsymbol{u}} \cdot \boldsymbol{n}\, d\Gamma_L - (F_{MI} - F_{AO}) \right) = 0 \\ \displaystyle\delta P_R \left( \int_{\Gamma_R} \dot{\boldsymbol{u}} \cdot \boldsymbol{n}\, d\Gamma_R - (F_{TR} - F_{PA}) \right) = 0 \end{cases} \Rightarrow \boldsymbol{G}_V\big(\{\boldsymbol{u}\}, \{\dot{\boldsymbol{u}}\}, \{\ddot{\boldsymbol{u}}\}, \{\boldsymbol{p}\}, P_L, P_R, F_{PA}, F_{MI}, F_{AO}, F_{AR}\big) = \boldsymbol{0} \quad \text{(S6.1)}$$

$$
\begin{cases}
F_{PA} - H(\bar{F}_{PA})\bar{F}_{PA} = 0 \colon \bar{F}_{PA} = \dfrac{1}{R_{PA}}\left(P_R - \dfrac{Q_{AP}}{C_{AP}}\right) \\
\dot{Q}_{AP} + F_{PA} + \dfrac{1}{R_{AP}}\left(\dfrac{Q_{AP}}{C_{AP}} - \dfrac{Q_{VP}}{C_{VP}}\right) = 0 \\
\dot{Q}_{PV} - \dfrac{1}{R_{AP}}\left(\dfrac{Q_{AP}}{C_{AP}} - \dfrac{Q_{VP}}{C_{VP}}\right) \\
\qquad + \dfrac{1}{R_{VP}}\left(\dfrac{Q_{VP}}{C_{VP}} - P_{LA}(e_{LA}, V_{LA})\right) = 0 \\
F_{MI} - H(\bar{F}_{MI})\bar{F}_{MI} = 0 \colon \bar{F}_{MI} = \dfrac{P_{LA}(e_{LA}, V_{LA}) - P_L}{R_{LA}} \\
\dot{V}_{LA} - \dfrac{1}{R_{VP}}\left(\dfrac{Q_{VP}}{C_{VP}} - P_{LA}(e_{LA}, V_{LA})\right) - F_{MI} = 0
\end{cases}
\Rightarrow \boldsymbol{G}_P\left(F_{PA}, Q_{AP}, \dot{Q}_{AP}, Q_{PV}, \dot{Q}_{PV} F_{MI}, V_{LA}, \dot{V}_{LA}, P_L, P_R\right) = \mathbf{0} \quad \text{(S6.2)}
$$

$$
\begin{cases}
F_{AO} - H(\bar{F}_{AO})\bar{F}_{AO} = 0 \colon \bar{F}_{AO} = \dfrac{1}{R_C}\left(P_L - \dfrac{Q_A}{C_A}\right) \\
\dot{Q}_A + F_{AO} + \dfrac{1}{R_A}\left(\dfrac{Q_A}{C_A} - \dfrac{Q_{VS}}{C_{VS}}\right) = 0 \\
\dot{Q}_{VS} - \dfrac{1}{R_A}\left(\dfrac{Q_A}{C_A} - \dfrac{Q_{VS}}{C_{VS}}\right) \\
\qquad + \dfrac{1}{R_{VS}}\left(\dfrac{Q_{VS}}{C_{VS}} - P_{RA}(e_{RA}, V_{RA})\right) = 0 \\
F_{TR} - H(\bar{F}_{TR})\bar{F}_{TR} = 0 \colon \ \bar{F}_{TR} = \dfrac{P_{RA}(e_{RA}, V_{RA}) - P_R}{R_{RA}} \\
\dot{V}_{RA} - \dfrac{1}{R_{VS}}\left(\dfrac{Q_{VS}}{C_{VS}} - P_{RA}(e_{RA}, V_{RA})\right) - F_{TR} = 0
\end{cases}
\Rightarrow \boldsymbol{G}_S\left(F_{AO}, Q_A, \dot{Q}_A, Q_{VS}, \dot{Q}_{VS} F_{TR}, V_{RA}, \dot{V}_{RA}, P_L, P_R\right) = \mathbf{0} \quad \text{(S6.3)}
$$

Here,$\{\boldsymbol{u}\}$, $\{\dot{\boldsymbol{u}}\}$, $\{\ddot{\boldsymbol{u}}\}$, and $\{\boldsymbol{p}\}$ denote the nodal values of the displacement, the velocity, the acceleration, and the hydrostatic pressure to be interpolated on the tetrahedral MINI(5/4c) elements, respectively. The variables of the system are assigned to the total unknown vector and its time derivative:

$$
\boldsymbol{U} = \begin{pmatrix} \boldsymbol{U}_V \\ \boldsymbol{U}_P \\ \boldsymbol{U}_S \end{pmatrix}, \quad \dot{\boldsymbol{U}} = \begin{pmatrix} \dot{\boldsymbol{U}}_V \\ \dot{\boldsymbol{U}}_P \\ \dot{\boldsymbol{U}}_S \end{pmatrix} \tag{S6.4}
$$

as

$$
\boldsymbol{U}_V = \begin{pmatrix} \{\boldsymbol{u}\} \\ \{\boldsymbol{p}\} \\ P_L \\ P_R \end{pmatrix}, \boldsymbol{U}_P = \begin{pmatrix} \overline{Q_P} \\ Q_{VP} \\ \overline{V_{LA}} \end{pmatrix}, \boldsymbol{U}_S = \begin{pmatrix} \overline{Q_A} \\ Q_{VA} \\ \overline{V_{RA}} \end{pmatrix} \tag{S6.5}
$$

and

$$\dot{\boldsymbol{U}}_V = \begin{pmatrix} \{\dot{\boldsymbol{u}}\} \\ \{\dot{\boldsymbol{p}}\} \\ \dot{P}_L \\ \dot{P}_R \end{pmatrix}, \dot{\boldsymbol{U}}_P = \begin{pmatrix} F_{PA} \\ \dot{Q}_P \\ \dot{Q}_{VP} \\ F_{MI} \\ \dot{V}_{LA} \end{pmatrix}, \dot{\boldsymbol{U}}_S = \begin{pmatrix} F_{AO} \\ \dot{Q}_A \\ \dot{Q}_{VA} \\ F_{TR} \\ V_{RA} \end{pmatrix} \tag{S6.6}$$

The missing components denoted by "$-$" in Equation (S6.5) do not affect the values of function:

$$\boldsymbol{G}(\boldsymbol{U}, \dot{\boldsymbol{U}}, \ddot{\boldsymbol{U}}) = \begin{pmatrix} \boldsymbol{G}_V(\boldsymbol{U}, \dot{\boldsymbol{U}}, \ddot{\boldsymbol{U}}) \\ \boldsymbol{G}_P(\boldsymbol{U}, \dot{\boldsymbol{U}}, \ddot{\boldsymbol{U}}) \\ \boldsymbol{G}_S(\boldsymbol{U}, \dot{\boldsymbol{U}}, \ddot{\boldsymbol{U}}) \end{pmatrix}. \tag{S6.7}$$

Thus, the missing components in $\boldsymbol{U}$ have no influence on the computational process, although they are also computed together with the other unknowns.

In the Newmark-beta time integration scheme, the variables $\boldsymbol{U}, \dot{\boldsymbol{U}},$ and $\ddot{\boldsymbol{U}}$ are updated from those at time $T$ to time $T + \Delta T$ so that both the interpolation relations in time (Equations (S6.8A-B)) and the equilibrium condition (Equation (S6.8C) at time $T + \Delta T$

$$\begin{cases} {}^{T+\Delta T}\dot{\boldsymbol{U}} = {}^{T}\dot{\boldsymbol{U}} + \Delta T\left(\gamma\, {}^{T+\Delta T}\ddot{\boldsymbol{U}} + (1-\gamma)\, {}^{T+\Delta T}\ddot{\boldsymbol{U}}\right) & \text{(S6.8A)} \\ {}^{T+\Delta T}\boldsymbol{U} = {}^{T}\boldsymbol{U} + \Delta T\, {}^{T}\dot{\boldsymbol{U}} + \Delta T^2\left(\beta\, {}^{T+\Delta T}\ddot{\boldsymbol{U}} + \left(\frac{1}{2} - \beta\right) {}^{T}\ddot{\boldsymbol{U}}\right) & \text{(S6.8B)} \\ \boldsymbol{G}\left({}^{T+\Delta T}\boldsymbol{U}, {}^{T+\Delta T}\dot{\boldsymbol{U}}, {}^{T+\Delta T}\ddot{\boldsymbol{U}}\right) = \boldsymbol{0} & \text{(S6.8C)} \end{cases}$$

are fulfilled. Here, $\beta$ and $\gamma$ are the interpolation weights which determine the numerical accuracy and stability of this scheme. In our case, we adopted $\beta = 0.3025$ and $\gamma = 0.6$.

To solve this system, Newton iterations are applied from the initial guess:

$${}^{T+\Delta T}\boldsymbol{U}^{(0)} \equiv {}^{T}\boldsymbol{U}, {}^{T+\Delta T}\dot{\boldsymbol{U}}^{(0)} \equiv {}^{T}\dot{\boldsymbol{U}}, {}^{T+\Delta T}\ddot{\boldsymbol{U}}^{(0)} \equiv {}^{T}\ddot{\boldsymbol{U}}. \tag{S6.9}$$

From Equations (S6.8A-B), the updates of $\dot{\boldsymbol{U}}$ and $\boldsymbol{U}$ at the first iteration are given from that of $\ddot{\boldsymbol{U}}$ as follows.

$$\begin{cases} \Delta\dot{\boldsymbol{U}}^{(1)} \equiv \Delta T\left({}^{T}\ddot{\boldsymbol{U}} + \gamma\Delta\ddot{\boldsymbol{U}}^{(1)}\right) \\ \Delta\boldsymbol{U}^{(1)} \equiv \Delta T \cdot {}^{T}\dot{\boldsymbol{U}} + \Delta T^2\left(\frac{1}{2}{}^{T}\ddot{\boldsymbol{U}} + \beta\Delta\ddot{\boldsymbol{U}}^{(1)}\right) \end{cases} \tag{S6.10}$$

The linearization of Equation (S6.8C) at the initial guess (Equation (S6.9)) with the increments in Equation (S6.10) gives the linear equation for $\ddot{\boldsymbol{U}}^{(1)}$:

$$\begin{aligned} \left(\boldsymbol{M}^{(0)} + \gamma\Delta T\boldsymbol{C}^{(0)} + \beta\Delta T^2\boldsymbol{K}^{(0)}\right)\Delta\ddot{\boldsymbol{U}}^{(1)} &= -\boldsymbol{G}\left({}^{T+\Delta T}\boldsymbol{U}^{(0)}, {}^{T+\Delta T}\dot{\boldsymbol{U}}^{(0)}, {}^{T+\Delta T}\ddot{\boldsymbol{U}}^{(0)}\right) - \boldsymbol{C}^{(0)}\left(\Delta T \cdot {}^{T}\ddot{\boldsymbol{U}}\right) \\ &\quad - \boldsymbol{K}^{(0)}\left(\Delta T \cdot {}^{T}\dot{\boldsymbol{U}} + \frac{1}{2}\Delta T^2 \cdot {}^{T}\ddot{\boldsymbol{U}}\right) \end{aligned} \tag{S6.11}$$

where the matrices are given by the derivatives of $\boldsymbol{G}$ , as follows ($k = 0$).

$$\begin{cases} \boldsymbol{M}^{(k)} \equiv \partial \boldsymbol{G}/\partial \ddot{\boldsymbol{U}} \left({}^{T+\Delta T}\boldsymbol{U}^{(k)}, {}^{T+\Delta T}\dot{\boldsymbol{U}}^{(k)}, {}^{T+\Delta T}\ddot{\boldsymbol{U}}^{(k)}\right) \\ \boldsymbol{C}^{(k)} \equiv \partial \boldsymbol{G}/\partial \dot{\boldsymbol{U}} \left({}^{T+\Delta T}\boldsymbol{U}^{(k)}, {}^{T+\Delta T}\dot{\boldsymbol{U}}^{(k)}, {}^{T+\Delta T}\ddot{\boldsymbol{U}}^{(k)}\right) \\ \boldsymbol{K}^{(k)} \equiv \partial \boldsymbol{G}/\partial \boldsymbol{U} \left({}^{T+\Delta T}\boldsymbol{U}^{(k)}, {}^{T+\Delta T}\dot{\boldsymbol{U}}^{(k)}, {}^{T+\Delta T}\ddot{\boldsymbol{U}}^{(k)}\right) \end{cases} \tag{S6.12}$$

For the first iteration, we may solve Equation (S6.11) to obtain $\Delta\ddot{\boldsymbol{U}}^{(1)}$, then we update the variables according to:

$$\begin{cases} {}^{T+\Delta T}\ddot{\boldsymbol{U}}^{(1)} \equiv {}^{T+\Delta T}\ddot{\boldsymbol{U}}^{(0)} + \Delta\ddot{\boldsymbol{U}}^{(1)} \\ {}^{T+\Delta T}\dot{\boldsymbol{U}}^{(1)} \equiv {}^{T+\Delta T}\dot{\boldsymbol{U}}^{(0)} + \Delta T\left({}^{T}\ddot{\boldsymbol{U}} + \gamma\Delta\ddot{\boldsymbol{U}}^{(1)}\right) \\ {}^{T+\Delta T}\boldsymbol{U}^{(1)} \equiv {}^{T+\Delta T}\boldsymbol{U}^{(0)} + \Delta T \cdot {}^{T}\dot{\boldsymbol{U}} + \Delta T^2\left(\frac{1}{2}{}^{T}\ddot{\boldsymbol{U}} + \beta\Delta\ddot{\boldsymbol{U}}^{(1)}\right) \end{cases} \tag{S6.13}$$

Although the initial guess in Equation (S6.9) does not necessarily fulfill the interpolation relations in Equations (S6.8A-B), the solution after the first iteration in Equation (S6.13) fulfills them, regardless of the accuracy of the solution in Equation (S6.11). Thus, the relationships between the updates after the first iteration become different from those in Equation (S6.10)

$$\begin{cases} \Delta\dot{\boldsymbol{U}}^{(k+1)} \equiv \Delta T\gamma\Delta\ddot{\boldsymbol{U}}^{(k+1)} \\ \Delta\boldsymbol{U}^{(k+1)} \equiv \Delta T^2\beta\Delta\ddot{\boldsymbol{U}}^{(k+1)} \end{cases} \quad k > 0 \tag{S6.14}$$

Thus, the linearized equation for $\Delta\ddot{\boldsymbol{U}}^{(k)}$ is

$$\left(\boldsymbol{M}^{(k)} + \gamma\Delta T\boldsymbol{C}^{(k)} + \beta\Delta T^2\boldsymbol{K}^{(k)}\right)\Delta\ddot{\boldsymbol{U}}^{(k+1)} = -\boldsymbol{G}\left({}^{T+\Delta T}\boldsymbol{U}^{(k)}, {}^{T+\Delta T}\dot{\boldsymbol{U}}^{(k)}, {}^{T+\Delta T}\ddot{\boldsymbol{U}}^{(k)}\right) \quad k > 0 \tag{S6.15}$$

and the solution is updated by

$$\begin{cases} {}^{T+\Delta T}\ddot{\boldsymbol{U}}^{(k+1)} \equiv {}^{T+\Delta T}\ddot{\boldsymbol{U}}^{(k)} + \Delta\ddot{\boldsymbol{U}}^{(k+1)} \\ {}^{T+\Delta T}\dot{\boldsymbol{U}}^{(k+1)} \equiv {}^{T+\Delta T}\dot{\boldsymbol{U}}^{(k)} + \Delta T\gamma\Delta\ddot{\boldsymbol{U}}^{(k+1)} \\ {}^{T+\Delta T}\boldsymbol{U}^{(k+1)} \equiv {}^{T+\Delta T}\boldsymbol{U}^{(k)} + \Delta T^2\beta\Delta\ddot{\boldsymbol{U}}^{(k+1)} \end{cases} \quad k > 0 \tag{S6.16}$$

In our simulation, the iterations were terminated when the residual (the right-hand side in Equation (S6.15)) was sufficiently small.

**S7. Parallel Performance in the Beating-heart Simulation**

Figure S7.1 compares the elapsed times for simulating 1 ms at the peak of the systolic phase for various numbers of cores, ranging from 240 to 3840. Here, 32 elements were assigned to each core in the minimal configuration (#cores=240), while 2 elements were assigned to each core in the maximal configuration (#cores=3840). During the computation required for 1 ms, calculation of the macroscale portion occurred 250 times, and required about 65 s, regardless of the numbers of cores used. Because the elapsed time for the macroscale part was sufficiently small, even in the maximal configuration, a further reduction can be expected if a much larger number of cores are available. Figure S7.1(B) shows the effectiveness of using a large macroscale time step size, $\Delta T$. If we choose a smaller time step, for example, $\Delta T = 312.5$ ns ($n = \Delta T/\Delta t = 1250$), the macroscale computational

portion occupies more than half of the time. Thus, an MTS scheme that admits a large macroscale time step size is necessary for this application.

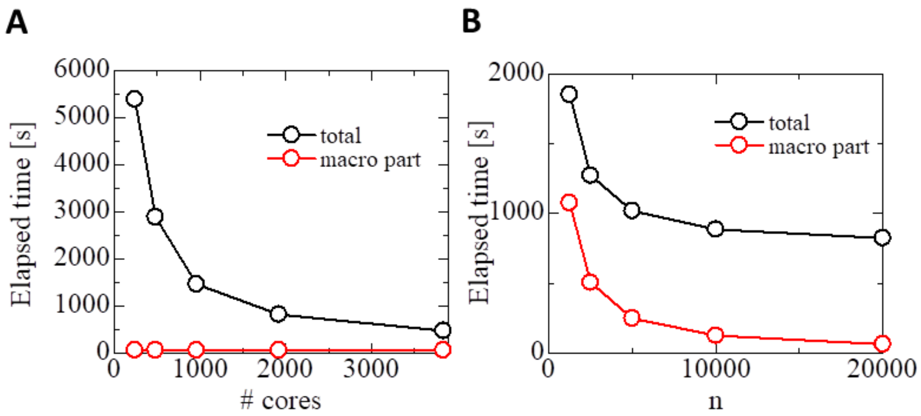


**Figure S7.1** Elapsed time per 1 ms duration at the peak of the systolic phase ($T = 0.225$ s). (A) The total elapsed time (black) and the elapsed time for the macro portion (red) are plotted for various numbers of cores (240, 480, 960, 1920, and 3840). The time step sizes are set at $\Delta t = 0.25$ ns and $\Delta T = 5000$ ns. (B) The total elapsed time (black) and the elapsed time for the macro portion (red) are plotted for various macroscale time step sizes ($n = \Delta T / \Delta t =$125, 250, 500, 1000, and 20000). The microscale time step size was set at $\Delta t = 0.25$ ns, and the number of cores was 1920.
